# Azoles as Auxiliaries and Intermediates in Prebiotic Nucleoside Synthesis

**DOI:** 10.1021/jacs.2c07774

**Published:** 2022-10-17

**Authors:** Dougal J. Ritson, Mikolaj W. Poplawski, Andrew D. Bond, John D. Sutherland

**Affiliations:** † MRC − Laboratory of Molecular Biology, Francis Crick Avenue, Cambridge Biomedical Campus, Cambridge CB2 0QH, U.K.; ‡ Yusuf Hamied Department of Chemistry, University of Cambridge, Lensfield Road, Cambridge CB2 0QH, U.K.

## Abstract

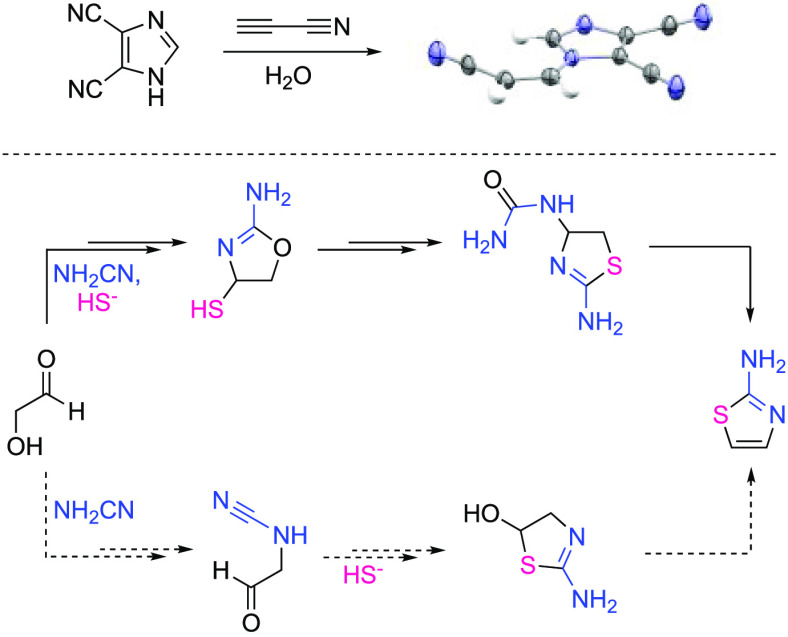

4,5-Dicyanoimidazole and 2-aminothiazole are azoles that have previously been implicated in prebiotic nucleotide synthesis. The former compound is a byproduct of adenine synthesis, and the latter compound has been shown to be capable of separating C_2_ and C_3_ sugars via crystallization as their aminals. We now report that the elusive intermediate cyanoacetylene can be captured by 4,5-dicyanoimidazole and accumulated as the crystalline compound *N-*cyanovinyl-4,5-dicyanoimidazole, thus providing a solution to the problem of concentration of atmospherically formed cyanoacetylene. Importantly, this intermediate is a competent cyanoacetylene surrogate, reacting with *ribo-*aminooxazoline in formamide to give *ribo-*anhydrocytidine — an intermediate in the divergent synthesis of purine and pyrimidine nucleotides. We also report a prebiotically plausible synthesis of 2-aminothiazole and examine the mechanism of its formation. The utilization of each of these azoles enhances the prebiotic synthesis of ribonucleotides, while their syntheses comport with the cyanosulfidic scenario we have previously described.

## Introduction

The abiogenic formation of the canonical nucleotides on early Earth is a problem that has intrigued and challenged chemists for decades. Compared to conventional synthetic organic chemistry, prebiotic synthesis is restricted by the number of reagents and starting materials that would have been available on early Earth. Adding to this problem is the fact that little is known for certain regarding the geologic and atmospheric state of Earth in the Hadean, which in turn would have impacted the types and abundances of available reagents. Exacerbating matters further, the nucleotides must have been selectively produced as the β-ribofuranosyl isomers with canonical regiospecific nucleobase attachment to allow competent Watson–Crick base-pairing in oligomeric materials. Cognizant of these requirements, we have attempted to offer a realistic rationale for the emergence of the canonical nucleotides over other potentially interfering isomers, and although a viable prebiotic route was suggested,^1^ and then improved,^2−4^ we felt that two of the steps (vide infra) were limiting, requiring particular environmental niches and/or the loss of potentially useful materials. Consequently, we wondered if there were as yet undiscovered reagents or pathways, which would broaden the scope of the geologic setting and streamline prebiotic nucleotide synthesis.

**Scheme 1:**
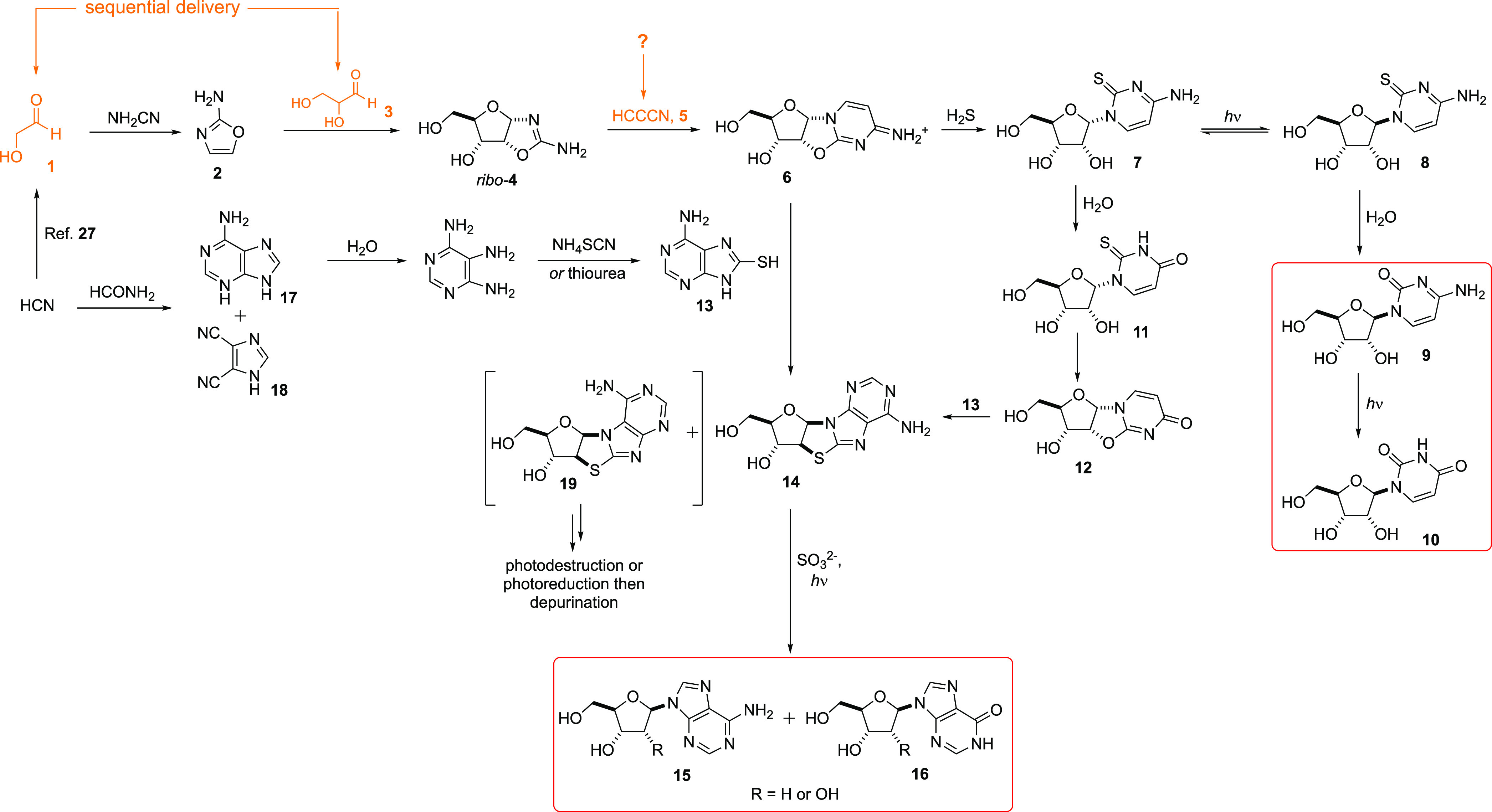
Prebiotic Synthesis of Purine and Pyrimidine Nucleosides (**9**, **10**, **15**, and **16**) through a Divergent Pathway Guanosine is not formed in this scheme; but inosine **16** is formed, which can act as a competent replacement for guanosine in nonenzymatic RNA copying. It should be noted that the 2′-deoxy congeners of **15** and **16** (R = H) are formed simultaneously with the ribonucleosides **15** and **16** (R = OH).

The optimal reaction sequence, as it stood, required the product of cyanamide (NH_2_CN) addition to glycolaldehyde **1**, 2-aminooxazole **2**, to react with glyceraldehyde **3**, thus forming a diastereomeric mixture of pentose aminooxazolines **4** (Scheme 1)^1^ — compounds that Sanchez and Orgel had previously made by condensation of the relevant sugar with NH_2_CN.^5^ Crucially, *ribo-***4** crystallizes from the mixture allowing the other diastereomers to be washed away,^6,7^ thereby ensuring the correct stereo- and furanosyl configuration of the eventual nucleosides.^2−4^ While the correct diastereoisomer can be selected at this point, it is of significant interest that *ribo-***4** also has the potential to be crystallized in an enantiopure form.^7,8^*Ribo-***4** can then undergo reaction with cyanoacetylene **5** to form *ribo-*anhydrocytidine **6**.^5^ This compound presents an opportunity for divergence in the synthesis of purine and pyrimidine nucleosides, undergoing reaction with hydrosulfide (optimally in formamide) to give α-thiocytidine **7**, which, after photochemical epimerization to the β-isomer **8**, can partially hydrolyze to cytidine **9** and uridine **10**.^2^ Remaining **7** hydrolyzes slowly affording α-thiouridine **11**; however, facile cyclization occurs and results in *ribo-*anhydrouridine **12**.^3^ Both **6** and **12** are competent glycosyl donors for reaction with 8-mercaptoadenine **13** in the dry state to give *N*9-8,2′-anhydro-thioadenosine **14**, which can be photochemically reduced to adenosine **15**, itself a precursor to inosine **16**, or the 2′-deoxy variants thereof.^3,4^ 8-Mercaptoadenine **13** can be conveniently obtained from adenine **17** (Scheme 1),^3^ and the clean formation of **17** and 4,5-dicyanoimidazole **18** from cyanide and formamide has also been demonstrated.^9,10^ While the *N*7*-*regioisomer of **14**, *N*7-8,2′-anhydro-thioadenosine **19**, is also formed in the glycosylation step, depending on the conditions of photoreduction, this compound is either photochemically destroyed or reduced to *N*7-2′-deoxyadenosine, which is hydrolytically labile. Thus, the two compounds that could have led to heterogeneity in nucleoside regio- and stereochemistry, **7** and **19**, are either recycled or expunged from the synthesis completely, thereby providing three of the four canonical nucleosides and a competent Watson–Crick base-pairing replacement for guanosine with strict adherence to the stereochemistry, nucleobase regioisomerism, and furanosyl configuration of sugars that are found in extant biology. This is of critical importance because the nonenzymatic incorporation of nucleotide monomers into a polymeric chain would be expected to be indiscriminate, and if the starting pool of mononucleotides contained heterogeneity in sugar configuration and/or stereochemistry and/or nucleobase regioconnectivity, the resulting polymer would be unable to align and take part in Watson–Crick base-pairing with a complementary strand of canonical RNA/DNA (with the exception of the *arabino-*furanosyl series). Although other prebiotic routes to RNA nucleosides and nucleotides have been suggested, ignoring their requirement for pure ribose or the plausibility of the syntheses in general, they either result in mixtures of α- and β-nucleosides with pyranosyl and furanosyl sugar configurations^11−13^ or contain regioisomeric mixtures of nucleobases and are produced in negligible amounts.^14^ Thus, the onus of proof is on those who deem such products plausible to show how the random polymerization of such mixtures can give rise to molecules with heritable information.

While the latter stages of the synthesis outlined in Scheme 1 have proven robust, with the pyrimidines being stable under conditions of the formation of purines,^3^ the first step in the synthesis demands sequential delivery of glycolaldehyde **1** and then glyceraldehyde **3**. Later in the proposed route to nucleosides described above, cyanoacetylene **5** is key, and, to date, there exists no other prebiotic route to the canonical pyrimidine nucleosides in significant yield that does not require **5**. Although only comprised of five atoms, the prebiotic synthesis of **5** is not trivial. The Cu^2+^ coupling of cyanide and acetylene to form **5** was demonstrated several years ago, but the requirement for stoichiometric copper likely means this route could only have been operational in particular environmental niches (given modern Earth′s crustal abundance of copper is ∼50 ppm), such as in areas of metallogenic enrichment occurring after a large impact with ensuing hydrothermal processing or differentiation of the melt, as evidenced today in locations such as the Sudbury Igneous Complex.^15−17^ Alternatively, if ultrareduced magmas existed on early Earth, it is theoretically possible that the delivery of **5** through surface hydrothermal systems could have occurred.^18^ The only proven, global means of producing cyanoacetylene **5** was reported by Sanchez et al., who showed that electrical discharge through a reduced atmosphere (containing CH_4_ + N_2_) resulted in the synthesis of **5**.^19^ More recently, high-energy UV irradiation, as would be experienced at high altitude, has also been shown to form **5** in a methane–dinitrogen atmosphere, though generally less efficiently than via electrical discharge.^20^ Earth′s primary atmosphere is expected to have been highly reduced, but it is widely accepted that this atmosphere would have been lost rapidly, being replaced by a mildly reduced or neutral atmosphere.^21,22^ However, perturbations in the atmospheric redox state would have occurred as a consequence of late accretion, when impacts from large objects containing a metallic iron core would have resulted in the production of copious amounts of H_2_ after the reaction of Fe^0^ with H_2_O.^23^ Larger impactors would result in more reduced atmospheres, which would persist for longer periods of time.^24^ Thus, the atmospheric synthesis of cyanoacetylene **5** during these epochs would have been viable, as is observed in the reduced atmosphere of Titan today.^20^ Nevertheless, any **5** formed in the atmosphere would only be present in negligible concentrations, which would have necessitated its concentration in groundwater to be of use for prebiotic chemistry; hard to envisage to any great extent given the modest boiling point of **5** (45 °C). Shapiro also noted that the instability of **5** with regard to various nucleophiles may hinder any potential accumulation or desirable reaction of **5**.^25^ For atmospherically produced cyanoacetylene **5** to have been useful for prebiotic chemistry, a protection step must have taken place, which presumably also allowed its concentration, a point that has not been addressed in other routes to nucleosides that invoke **5**. The masking group in question was likely related to the prebiotic route for nucleoside synthesis, such that at any location where the types of chemistry required for nucleoside synthesis were occurring, the accumulation of (a derivative of) **5** could also be expected. Thus, 4,5-dicyanoimidazole **18** (DCI), the major byproduct of prebiotic adenine **17** synthesis,^10^ seemed an interesting nucleophile to investigate given its low p*K*_a_ and crystalline nature when uncharged.

## Results and Discussion

**Scheme 2:**
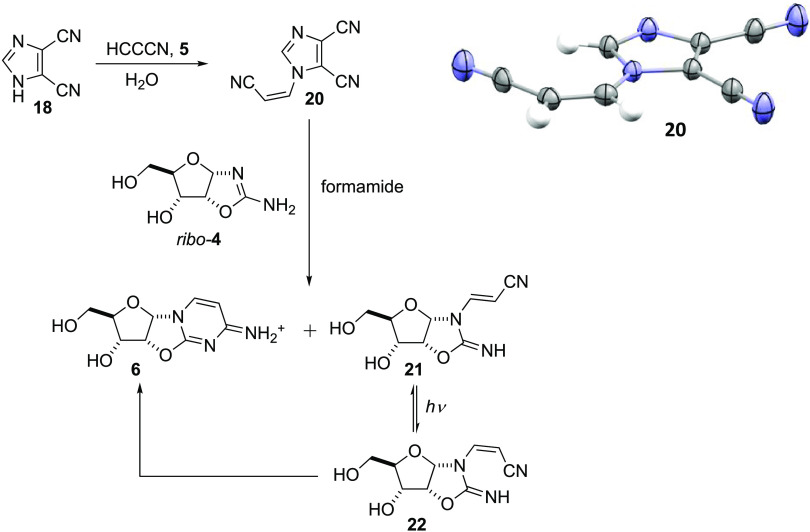
Prebiotic Formation of CV-DCI **20** and Its Conversion to the Purine and Pyrimidine Precursor *ribo-A*nhydrocytidine **6**

Following the line of reasoning outlined above, 4,5-dicyanoimidazole **18** was suspended in H_2_O/D_2_O and the pH was adjusted to 5.2, after which a solution of cyanoacetylene **5** in H_2_O was added. The suspension soon dissolved, and the reaction was followed by ^1^H NMR spectroscopy, where clean addition of **18** to **5** was observed to take place on a timescale of hours (Figures S1–S3). The following morning, crystals had formed and single-crystal X-ray analysis determined the structure to be that of *N-*cyanovinyl-4,5-dicyanoimidazole **20** (CV-DCI, Scheme 2). When a sample of **20** was prepared and a portion recovered and dried, no change to the composition of **20** was observed after storing the solid for 6 months on the bench under standard atmospheric conditions (Figure S4). When **20** was mixed in water with various nucleophiles, such as cyanide, phosphate, adenosine, and ammonia, little reaction, if any, was observed (Figures S5–S7). Although a means for the protection, purification, and accumulation of a cyanoacetylene analogue appeared to have been found, partially alleviating Shapiro′s concerns,^25^ it did raise the question of the recovery of cyanoacetylene **5** for further synthesis, as the reaction of **20** with *ribo*-aminooxazoline *ribo-***4** in water gave low conversion to **6**, possibly due to the low solubility of both compounds in water. Considering that the thiolysis of *ribo-*anhydrocytidine **6** takes place most effectively in formamide, or wet formamide, the dissolution of **20** in formamide for reaction with *ribo-***4** (itself only sparingly soluble in water) would be consistent with the proposed reaction sequence (Scheme 1).^2^ Thus, **20** (200 mM) was dissolved in formamide, *ribo-***4** (100 mM) was added, and the mixture was warmed to 50 °C. Clean reaction took place, with the anhydro intermediate **6** forming in ∼50% yield after 2 days, and the only other discernible product appeared to be the *trans*-cyanovinylated product **21** (∼14%, Scheme 2 and Figures S8 and S9). Prolonged reaction at 50 °C, or heating the reaction to 90 °C, did not convert **21** to **6** (Figure S10). However, upon irradiation of the crude reaction mixture, either in neat formamide or after dilution in water, *E*/*Z*-isomerization appeared to have occurred, so allowing cyclization of the imino nitrogen of photoisomer **22** onto its nitrile group, as the yield of **6** had increased from ∼48 to ∼55% in neat formamide or to ∼58% when diluted in water (Scheme 2 and Figure S11). While CV-DCI **20** seems to be an excellent candidate for the concentration, purification, and protection of atmospherically produced cyanoacetylene **5**, there are other potential candidates that could also fulfill this role. A comprehensive survey of possible prebiotic masking groups was not made, but at least one potential solution to the cyanoacetylene problem had been found, which was consistent with the pathway outlined in Scheme 1, and consequently, our attention turned to the issue of the separation of glycolaldehyde **1** and glyceraldehyde **3**, which should allow the most efficient synthesis of *ribo*-aminooxazoline *ribo-***4** (Scheme 1).

Cyanide is heavily implicated in the chemistry depicted in Scheme 1 (the synthesis of adenine **17**, DCI **18** and cyanoacetylene **5**, and thiocyanate and thiourea, useful precursors to NH_2_CN, are derived from HCN/HS^–^ chemistry), but cyanide has traditionally been viewed as incompatible with sugars in prebiotic chemistry due to the immediate formation of cyanohydrins. However, we have previously reported the prebiotic synthesis of the sugars **1** and **3** through a photochemical Kiliani–Fischer-like process, which allows homologation of aldoses through the addition of cyanide and reduction of the resulting cyanohydrins by hydrogen atoms or hydrated electrons, themselves the products of UV irradiation of inorganic sulfur anions.^26−28^ Thus, glyceraldehyde **3** can be produced sequentially from glycolaldehyde **1**. Notwithstanding, the addition of NH_2_CN to **1** with the subsequent addition of **3** is still required for the highest-yielding synthesis of the aminooxazoline *ribo-***4**, and we have previously suggested that this could have been achieved in a flow system. According to this scenario, **1** is formed in one stream, which also contains NH_2_CN, before a confluence mixes **3**, formed in a separate stream, with **2**, the product of **1** + NH_2_CN.^29,30^ As it is likely that when **3** is formed some **1** would remain, a means to separate **1** and **3** from a mixture of the two would be very attractive and potentially reduce the geological model to one stream or body of water.

**Scheme 3:**
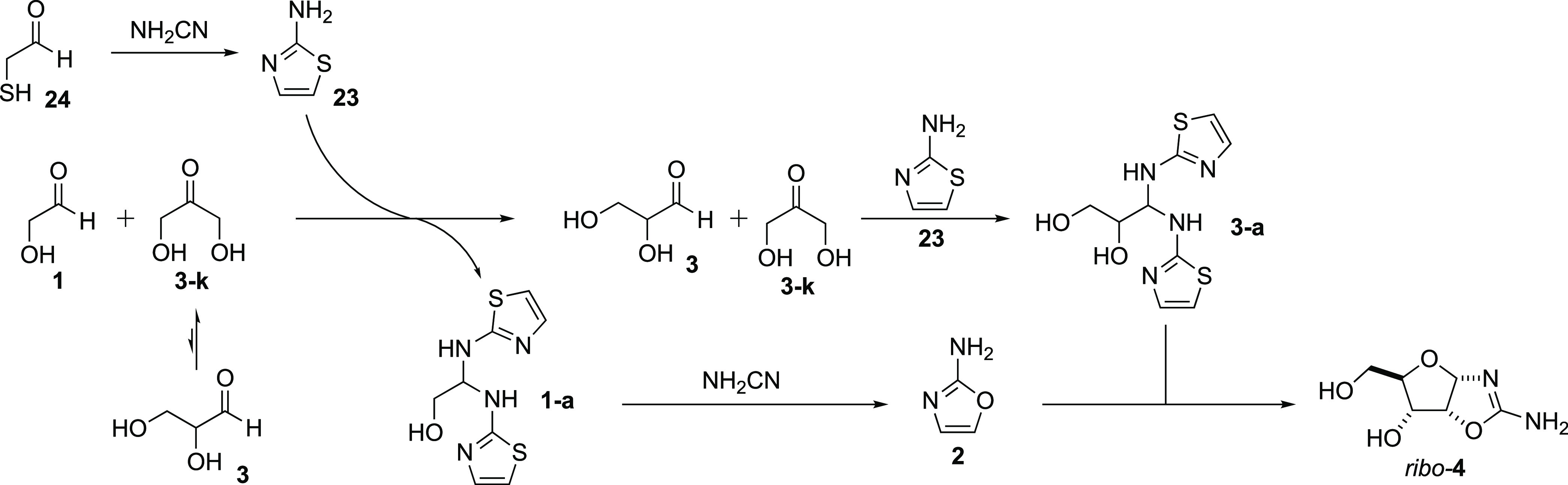
Potential Prebiotic Separation of C_2_ and C_3_ Sugars through the Preferential Formation of **1-a** over **3-a** While **3-k** is the preferred triose isomer at equilibrium (∼9:1), **23** will only form an aminal with the aldehyde carbonyl of **3**, and thus, **3-a** accumulates and crystallizes at the expense of **3-k**.

Islam et al. reported an elegant solution to this problem exploiting the crystalline properties of 2-aminothiazole aminals **1-a** and **3-a**, in particular, the thermodynamic preference for the formation of **1-a** over **3-a** and for the ability of the formation of **3-a** to overturn the preferred triose equilibrium of **3-k** over **3** (Scheme 3).^31^ While the downstream chemistry was demonstrated to be robust (rapid crystallization of **1-a** and the delayed crystallization of **3-a** allowing the separation of pure aminals, the reaction of crystallized **1-a** with NH_2_CN to form **2**, and the reaction of **2** with crystallized **3-a** to form *ribo*-**4**; see Scheme 3), the prebiotic synthesis of 2-aminothiazole **23** employed mercaptoacetaldehyde **24**. Although **24** has been suggested as a product of atmospheric chemistry, the conditions required an atmosphere composed of ∼50% ethane and ∼10% H_2_S, which is hard to reconcile with current thoughts concerning primitive Earth′s atmosphere, or even a transiently reduced one.^32^ Thus, a more plausible synthesis of **23** was required.

**Scheme 4:**
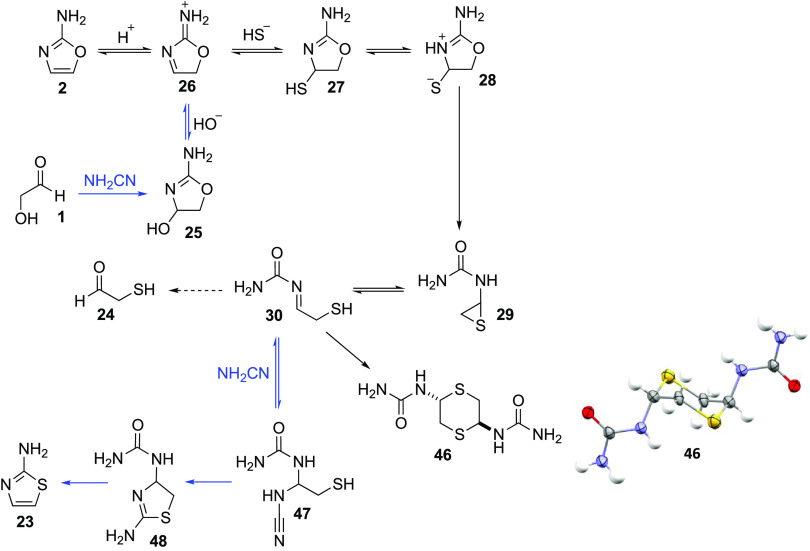
Potential Prebiotic Route to Mercaptoacetaldehyde **24** from 2-Aminooxazole **2** (Black Arrows) and the Eventual Synthesis of 2-Aminothiazole **23** from Glycolaldehyde **1** (Blue Arrows) Unidirectional arrows are used to indicate equilibria, which are expected to lie heavily, if not completely, to one side. The conversion of **28** to **29** would be expected to be unidirectional, given the reverse reaction requires a weak nucleophile to add to a poor electrophile. The condensation of urea with **24** can occur in principle but will be disfavored when the reaction is run in water under dilute conditions.^33^

The simplest means of achieving this would be to find a prebiotic route to **24**, given the efficiency of the synthesis of **23** from **24** and NH_2_CN.^31^ A conventional synthesis of **24** would have constituted the displacement of bromide from α-bromoacetaldehyde (or an equivalent) by thioacetamide, followed by hydrolysis of the resultant thioimidate. As organobromine compounds are not considered to have been prebiotically available, an alternate route must have existed. Glycolaldehyde **1** seems to be an obvious starting point, given the correct oxidation levels of the carbon atoms and that **1** can be made in good yield by cyanosulfidic photochemistry, which suggests that sulfur species would have been available at the time of the synthesis of **1**. However, the alcohol group of **1** is insufficiently reactive to undergo direct S_N_2 displacement by a thiol. As NH_2_CN is required, at least constitutionally, to form **23**, we wondered about the product of NH_2_CN and **1**, namely, 2-aminooxazole **2**. Ostensibly, **2** is unreactive toward nucleophiles, but the existence of the hydrate of **2** (**25**) at equilibrium suggested the availability of an iminium ion **26** for interception by nucleophiles (Scheme 4). Addition of HS^–^ would give the hemithioaminal **27**, and the zwitterion form **28** enforces the proximity of an excellent nucleophile and a leaving group. We therefore speculated that the episulfide **29** would result, and after ring-opening and hydrolysis of **30**, access to **24** could be achieved.

In the event, incubation of 2-aminooxazole **2** (100 mM) with NaSH (100 mM) in phosphate buffer (pH 7, 200 mM) at 40 °C for 24 h gave a solution containing microcrystals, and clean production of two new species which possessed similar ABX systems in the ^1^H NMR spectrum of the reaction mixture could be observed (Figure S12). It was initially assumed that **27** had reacted with the iminium ion **26**, leading to a pair of diastereomeric thioethers. These compounds did not give rise to **24** upon further heating.

**Scheme 5:**
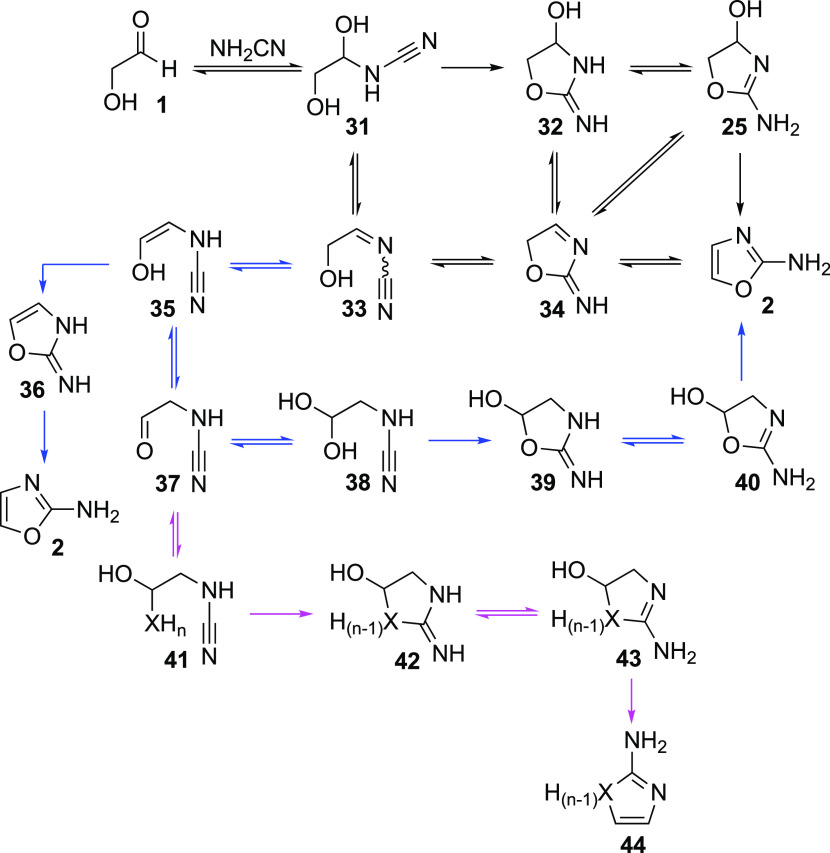
Proposed Mechanistic Routes for the Formation of **2** from **1** and NH_2_CN, and a Possible Avenue to Divert the Reaction Pathway to Azoles **44** Unidirectional arrows are used to indicate equilibria, which are expected to lie heavily, if not completely, to one side.

We then began to consider the mechanism of formation of **2** using **1** and NH_2_CN to see if an opportunity existed where sulfur could be incorporated into the forming heterocycle. The originally proposed mechanism for the synthesis of **2** runs along the lines of that depicted in Scheme 5 (black arrows);^1^ however, there exists the possibility of α-deprotonation of imine **33** leading to enamine **35** (Scheme 5, blue arrows). The enamine **35** can cyclize directly to **36** giving **2**, or hydration of the aldehyde tautomer of **35**, **37**, leads to **38**, which can now cyclize onto the cyanamide nitrile, thereby affording **39** and the thermodynamically preferred isomer **40** and ultimately **2**. If this reaction manifold were open, then aldehyde **37** would be susceptible to the addition of nucleophiles to give hemihydrates **41**, cyclization of which would provide **42** and finally azoles **44** (Scheme 5, magenta arrows). Although it has been suggested that 2-aminoimidazole **45** could form in such a manner, no evidence has been presented in favor of or against such a mechanism.^34^

Initially, we allowed glycolaldehyde **1** (100 mM) and NH_2_CN (150 mM) to react in the presence of NaSH (150 mM) in phosphate (200 mM, pH 7.0) or bicarbonate (200 mM, pH 9.2) buffer at 60 °C and used ^1^H NMR spectroscopy to monitor the reaction. Although **1** is supplied in its dimeric form, in dilute aqueous solution in the presence of an acid–base catalyst, it rapidly hydrolyzes to form glycolaldehyde and its hydrate. For example, in 200 mM phosphate buffer at pH 6.6, by routine ^1^H NMR spectroscopy, there is ∼90% of glycolaldehyde hydrate present in <10 min. Indeed, the rate to form equilibrium positions of the various monomers and dimers of **1** in water has already been reported.^35^ In phosphate buffer, 2-aminothiazole **23** and 2-aminooxazole **2** were produced in a ∼1:1 ratio, respectively, after 24 h reaction, but after 7 days, **23** was formed in ∼10% yield and the amount of **2** had been drastically reduced (∼1%, Figure S13). In bicarbonate buffer, the reaction was more selective for production of **23** over **2**, giving ∼9% of **23** after 24 h and ∼1% of **2** (Figure S14). The major product, however, was the same pair of diastereoisomers observed from the reaction of **2** with NaSH (Figures S12 and S14), but this time, the crystalline product was suitable for X-ray analysis. Single-crystal X-ray diffraction revealed that dithiane **46**, existing in the *trans-*diaxial form in the crystal lattice, was in fact the mystery compound (Scheme 4), and dissolution of the crystals in DMSO showed the equilibration of *trans-***46** with *cis-***46** on a timescale of hours (Figure S15). We attempted to reduce dimerization to a minimum by keeping the concentration of **1** low (≤25 mM), and in phosphate buffer at 60 °C, **23** could be formed from **1** (25 mM), NaSH (100 mM), and NH_2_CN (75 mM) in ∼10% yield after 3 days or in ∼14% yield after 7 days (Figure S16). At higher pH (9.2) using bicarbonate buffer, a much cleaner reaction was observed and **23** was formed in ∼26% yield after 3 days or ∼30% after 7 days (Figure S17). However, maximal yields were obtained after longer reaction times, which varied from 10 to 25 days and gave **23** in 32–40% yield. When a solution of **23** (100 mM) at pH 6.5 was evaporated at room temperature under a stream of N_2_ to the point of crystallization, <10% of **23** had been lost to evaporation (succinate used as an internal reference), suggesting that **23** could have been concentrated in groundwater.

With an efficient prebiotic synthesis of 2-aminothiazole **23** in hand, we then considered the mechanism of its formation. ^13^C-labeling studies revealed the connectivity of NH_2_CN to glycolaldehyde and suggested that the pathway outlined in Scheme 5 (the magenta pathway) may be correct (Figures S18–S21), but an inconsistency in the rate of product formation coupled with the observation of small amounts of dithiane **46** made us reconsider the mechanistic pathway. Under our optimal conditions for the formation of 2-aminothiazole **23** in bicarbonate buffer, at timepoints of 1, 2, and 3 h, the yield of **23** was 9, 10, and 10%, respectively. There was then a slow increase in the yield of **23** to reach a maximum yield of ∼40% after ∼25 days, which coincided with the decrease of another, unidentified species observed in the ^1^H NMR spectrum, and we thus conclude that at least two pathways to **23** are operational. The unidentified compound displayed a similar ^1^H NMR spectrum to that of dithiane **46**, and as **46** is derived from imine **30** (Scheme 4), we wondered if the unknown compound was the product of reaction of NH_2_CN and **30**, in which case the major pathway to **23** would follow that outlined in Scheme 4 (blue arrows) and the unidentified compound would be thiazoline **48**. When ^13^C-labeled cyanamide was employed in the same reaction, the intermediate assumed to be **48** displayed complex coupling patterns that could only be accounted for if more than one NH_2_^13^CN molecule had been incorporated and was consistent with the structure of **48** (Figure S22). Furthermore, when a sample of the crude reaction after 7 days of heating was subjected to mass spectrometry (ESI(+)), mass signals corresponding to [M + H]^+^, [M + Na]^+^, and [M_2_ + Na]^+^ for thiazoline **48** were found (Figure S23). This suggests that the minor and more rapid route to **23** is consistent with that first proposed, i.e., Scheme 5, magenta arrows (X = S, *n* = 1) and, if correct, also implies that the addition of NH_2_CN to **1** with ensuing cyclization is more rapid than the Amadori rearrangement of imine **33** to aldehyde **37** (Scheme 5). Whether ring closure occurs from hemi-aminal **31** to **32**, imine **33** to **34**, or from enamine **35** to **36** cannot be inferred, but *trans-***33** would be expected to be favored over *cis-***33**, and even more so to satisfy the intramolecular hydrogen bond donor–acceptor pair, thus cyclization of **33** to **34** should be inhibited. The inference then is that the originally proposed route to 2-aminooxazole **2** (Scheme 5, black arrows) is the major pathway followed, although a minor contribution from the second pathway (Scheme 5, blue arrows) can also be expected. Although the formation of **2** in H_2_^18^O resulted in ∼6% ^18^O incorporation into the heterocycle product, it cannot be conclusively determined if this is due to the pathway outlined in Scheme 5 (magenta arrows, X = O, *n* = 1) or from the exchange of carbonyl ^16^O in **1** with ^18^O from the solvent (via the hydrate of **1**) and ensuing isomerization giving glycolaldehyde-2-^18^O, which can then enter the major reaction manifold (Scheme 5, black arrows), or a combination of both. Similarly, the isotopic labeling in 2-aminoimidazole **45** (X = N, *n* = 2) when formed from ^15^NH_4_Cl, glycolaldehyde-1-^13^C and NH_2_CN were uninformative due to the fact that glycolaldehyde-1-^13^C was found to isomerize to glycolaldehyde-2-^13^C under the reaction conditions in the absence of NH_2_CN.

## Conclusions

**Scheme 6:**
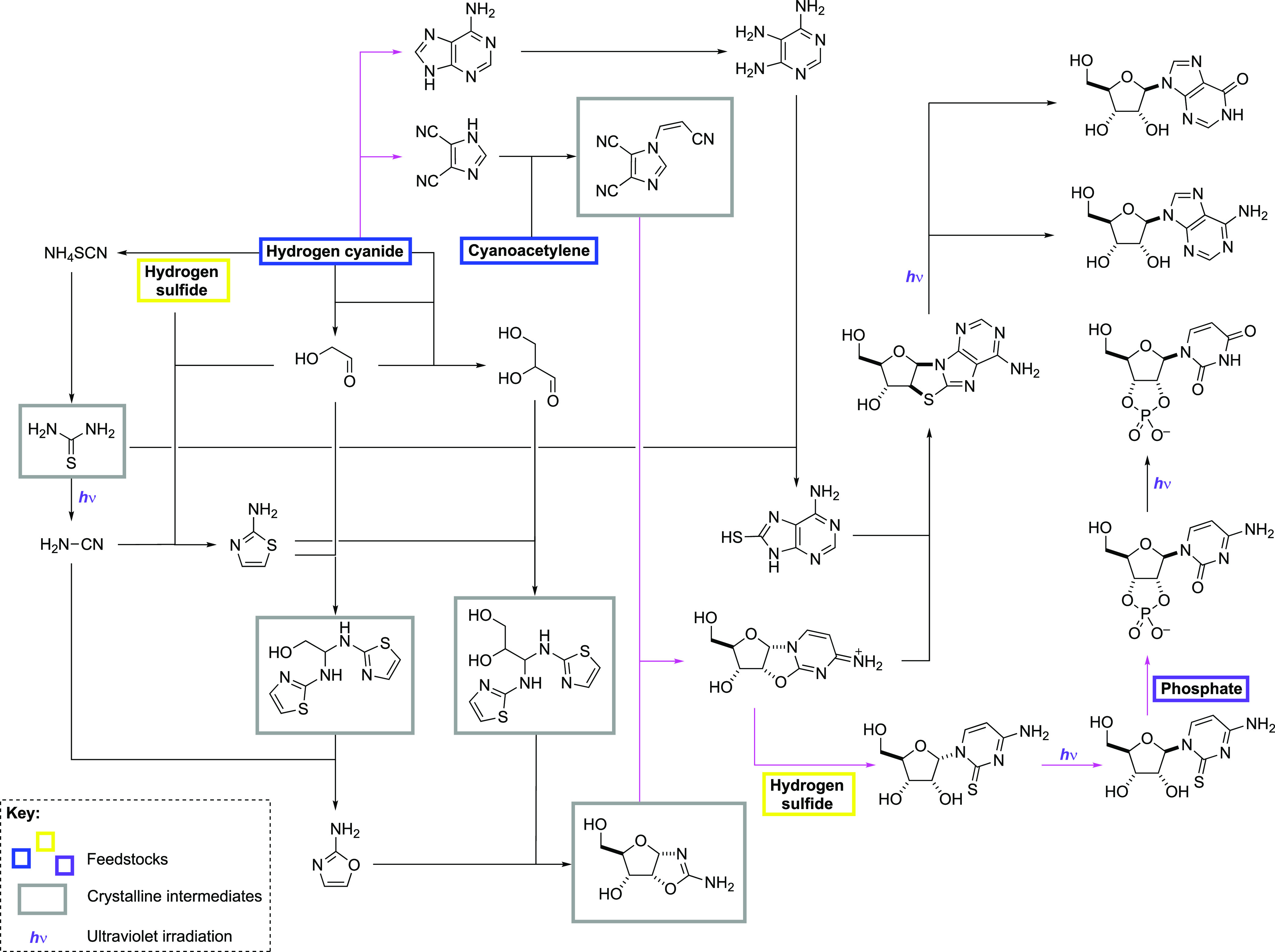
Modified Prebiotic Scheme, Highlighting the Simple Feedstocks Required and the Crystalline Intermediates through Which the Syntheses Proceed Black arrows depict reactions taking place in water, and magenta arrows depict reactions taking place in formamide.

Our overall scheme for the synthesis of prebiotic nucleosides has now evolved and includes the synthesis of 2-aminothiazole **23** from glycolaldehyde **1**, HS^–^, and NH_2_CN; the crystallization and separation of glycolaldehyde **1** and glyceraldehyde **3** as their aminals, **1-a** and **3-a**, respectively; and capture of cyanoacetylene **5** by DCI **18** as the crystalline derivative CV-DCI **20** (Scheme 6). It is noteworthy that thiourea, a precursor to cyanamide and reagent for 8-mercaptoadenine synthesis **13**, and *ribo-*aminooxazoline *ribo-***4** are also crystalline intermediates (Scheme 6).

To achieve high-yielding steps in prebiotic synthesis, the stepwise addition or separation of (at least some) reagents and/or reactions is required to avoid countless indiscriminate and unselective reactions taking place. Although the issue is sometimes conveniently sidestepped and suggestions even made to the contrary, there is a consensus that can be clearly discerned from the examination of literature experimental procedures — efficient and high-yielding prebiotic chemistry, just like conventional synthetic chemistry, needs sequential reagent addition, occasional purification steps, and the separation of certain reagents and conditions. In a planetary setting, we have suggested that the sequential addition of reagents could have been achieved through the confluence of streams carrying differing solutes.^29,30^ While this may have provided the opportunity for the addition of fresh reagent(s), as the reaction sequence progressed, byproducts would build up, and this indicates some type of purification process was necessary. In the proposed prebiotic scheme (Scheme 6), crystallization can take place at five junctures, meaning that soluble byproducts can be naturally washed away, and the clock is effectively reset for the next stage of synthesis from the pure, crystalline material.

## Supporting Information Available

General experimental information; compound characterization; supplementary figures and crystallographic data (PDF)
