# Supplementary material for: Azoles as Auxiliaries and Intermediates in Prebiotic Nucleoside Synthesis
Source: J Am Chem Soc. 2022 Oct 17;144(42):19447–55. doi: 10.1021/jacs.2c07774 (PMC9619408; doi:10.1021/jacs.2c07774)
Supplement: Supplementary file 1 — ja2c07774_si_001.pdf [file ja2c07774_si_001.pdf]

Supporting information for

Azoles as auxiliaries and intermediates in prebiotic nucleoside synthesis

Dougal J. Ritson[1], Mikolaj W. Poplawski[2], Andrew D. Bond[2], John D. Sutherland[1*]

Email: johns@mrc-lmb.cam.ac.uk

1) MRC – Laboratory of Molecular Biology, Francis Crick Avenue, Cambridge Biomedical Campus, Cambridge, CB2 0QH, U.K.
2) Yusuf Hamied Department of Chemistry, University of Cambridge, Lensfield Road, Cambridge, CB2 1EW, U. K.

Table of contents

General experimental 1

General procedures 2

Figures S1 – S23 3 – 25

X-Ray crystallographic data and Figures S24 and S25 26 – 27

References 28

General experimental

Reagents and solvents were bought from Sigma-Aldrich, Alfa Aesar and Santa Cruz Biotechnology and were used without further purification. ^{13}C-Labelled reagents had a ^{13}C-incorporation of 95 atom % or more. Reagents were weighed using a Sartorius AX124 M-Pact analytical balance and small volumes were measured using a Gilson™ Pipetman™. A Mettler Toledo SevenMulti pH/mv module fitted with a Thermo Scientific Orion 8103BN pH probe was used to measure pH, and deoxygenation of solvents and HCl/NaOH solutions was achieved by sparging with Ar for 20-30 min before use. Although deoxygenation of HCl and NaOH solutions, used to adjust the pH of reactions, may have altered the concentrations of these reagents, it was not deemed to be important as adjustment of the pH of the reaction the only consideration. Rigorous exclusion of O_2 from solutions after sparging was not possible, particularly when checking/adjusting the pH, where the solution would typically be exposed to the atmosphere for ~ 45 s. ^{1}H, ^{31}P And ^{13}C NMR spectra were acquired using a Bruker Ultrashield™ 400 Plus (at 400.1, 162.0 and 100.6 MHz, respectively) or alternatively, ^{1}H and ^{31}P NMR spectra were recorded using a Bruker Ascend™ 400 (at 400.2 and 162.0 MHz, respectively) using solvent suppression to collect ^{1}H NMR data if reactions were run in a D_2O/H_2O mixture. If spectra were unsatisfactory, a small amount of D_2O was added to the NMR sample and the spectrum reacquired. Yields were determined by relative integration of signals in ^{1}H or ^{31}P NMR spectra, or by addition of a known volume and concentration of a standard and relative integration to this signal. Coupling constants (*J*) are given in Hertz (Hz) and the notations d, t and q represent the multiplicities doublet, triplet and quartet. Chemical shifts (δ) are given in ppm. Mass spectra were acquired on an Agilent 1200 54 LC-MS system equipped with an electrospray ionization (ESI) source and a 6130 quadrupole spectrometer (LC solvents: A, 0.2% formic acid in H_2O; B, 0.2% formic acid in acetonitrile).

General procedures:

Cyanoacetylene ***5*** *preparation*

Following the procedure of Xiang *et al.*[1] Oven-dried sand (14.5 g) was ground with propiolamide (2.0 g, 28.9 mmol) then P_2O_5 (6.2 g, 43.5 mmol) added and quickly ground further before being charged to a twin-necked round-bottomed flask (250 mL) and spread around the sides. N_2 Was flushed through the system as a carrier gas, which passed through two cool traps, cooled to – 78 °C, then scrubbed through NaOH/bleach. The dry-state reaction was lowered into an oil bath heated at 135 °C and left for 2 h. The traps were warmed to 0 °C and washed with H_2O (13.03 g), the final mass of the cyanoacetylene solution was 13.323 g (~ 20% yield) which was split into batches and frozen at – 32 °C.

Preparation of cyanovinyl 4,5-dicyanoimidazole ***20***

4,5-Dicyanoimidazole **18** (10 mg, 0.085 mmol) was suspended in H_2O (200 mL) and 1 M NaOH added dropwise with mixing until all the solid had dissolved. A further portion (225 mg, 1.91 mmol) of **18** was suspended in cyanoacetylene solution (~ 0.4 M, 3 mL) and the imidazolate solution added to it. The reaction was sealed and gently tumbled for 3 d, after which the pH of the liquid phase was adjusted to ~ 7 with NaOH. The suspension was then cooled to 4 °C for 6 h and the mixture separated by centrifugation. The supernatant was removed and discarded, the solid was resuspended in H_2O and lyophilised. This gave the title compound as a near pure, off-white solid (171 mg, 1.01 mmol, see Figure S2 and S3).

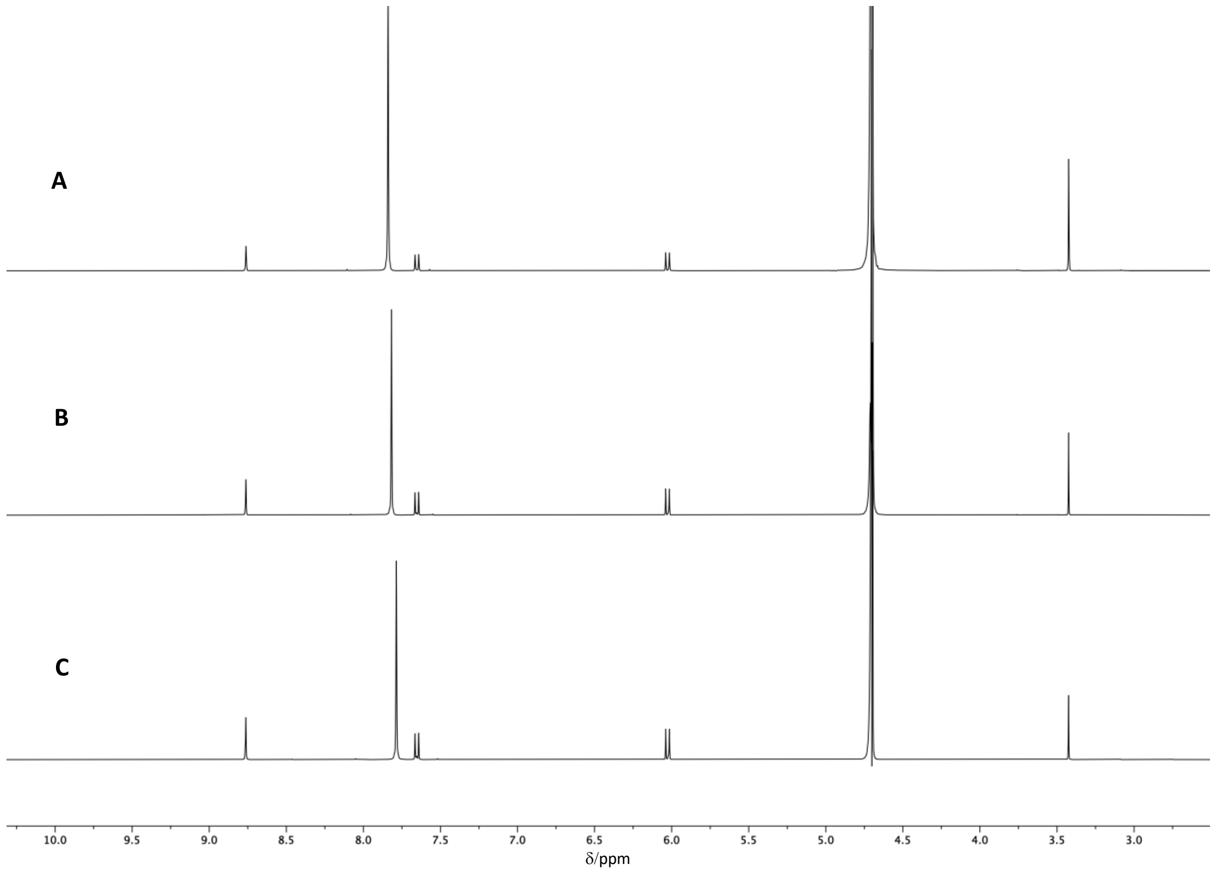

Figure S1 Reaction of 4,5-dicyanoimidazole **18** with cyanoacetylene **5**. 4,5-Dicyanoimidazole **18** (24 mg, 0.200 mmol) was suspended in H_2O/D_2O (0.4/0.1 mL) in an Eppendorf tube and the pH was adjusted to 5.3 with NaOH. A solution of **5** in H_2O (~ 0.4 M, 0.5 mL) was then added, and a portion of the reaction was transferred to an NMR spectroscopy tube and spectra acquired periodically after the solid had dissolved. In the morning crystals were recovered from the Eppendorf tube and analysed by NMR spectroscopy (see Figure S2) and X-ray diffraction. A – ^{1}H NMR spectrum of the reaction after 1.5 h; B – ^{1}H NMR spectrum of the reaction after 6 h; C – ^{1}H NMR spectrum of the reaction after 12 h.

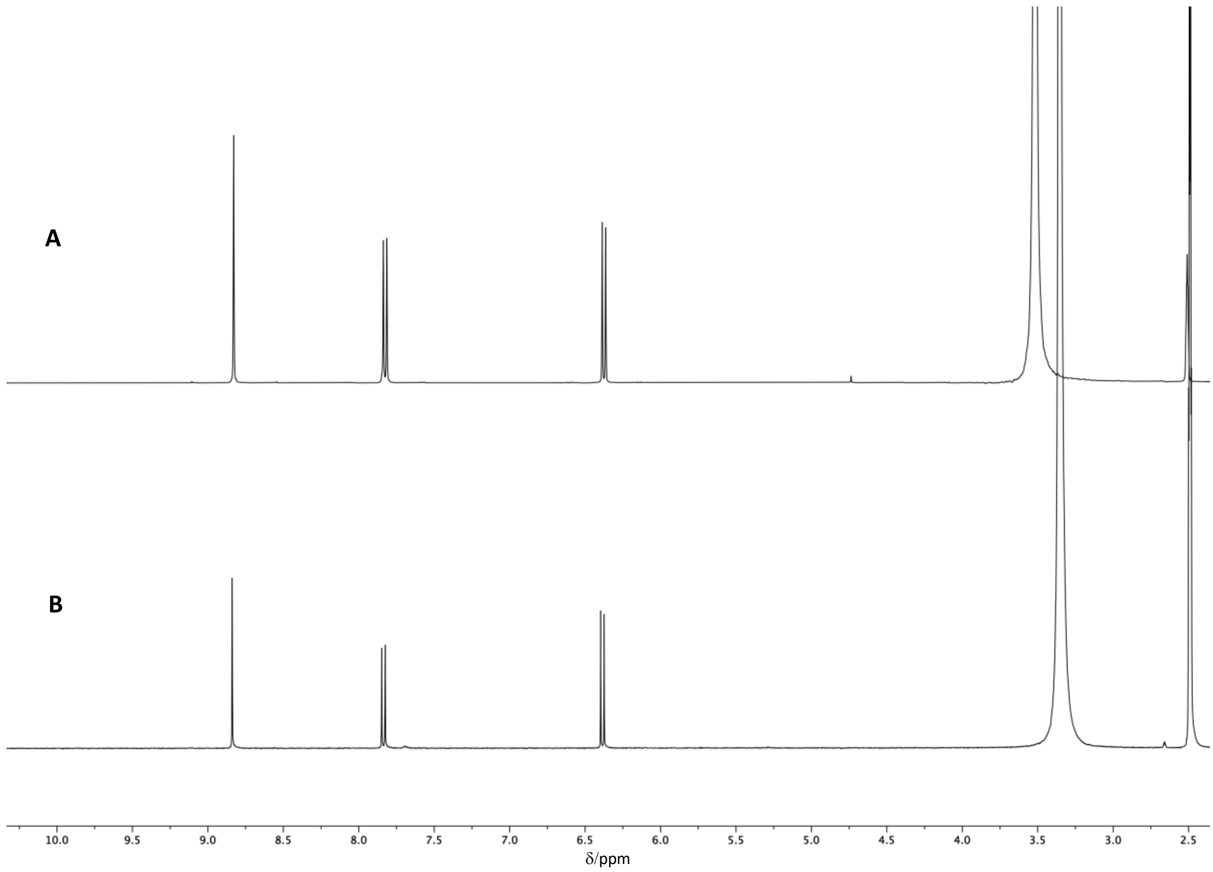

Figure S2 ^{1}H NMR spectra of cyanovinyl 4,5-dicyanoimidazole **20**. A – ^{1}H NMR spectrum of the crystals isolated from the procedure of Figure S1, run in DMSO-d_6; B – ^{1}H NMR spectrum of the crystals isolated following preparation of cyanovinyl 4,5-dicyanoimidazole **20** (see General Procedures). (DMSO-d_6) δ 8.85 (s, 1 H), 7.84 (d, *J* = 9.2, 1 H), 6.39 (d, *J* = 9.2, 1 H).

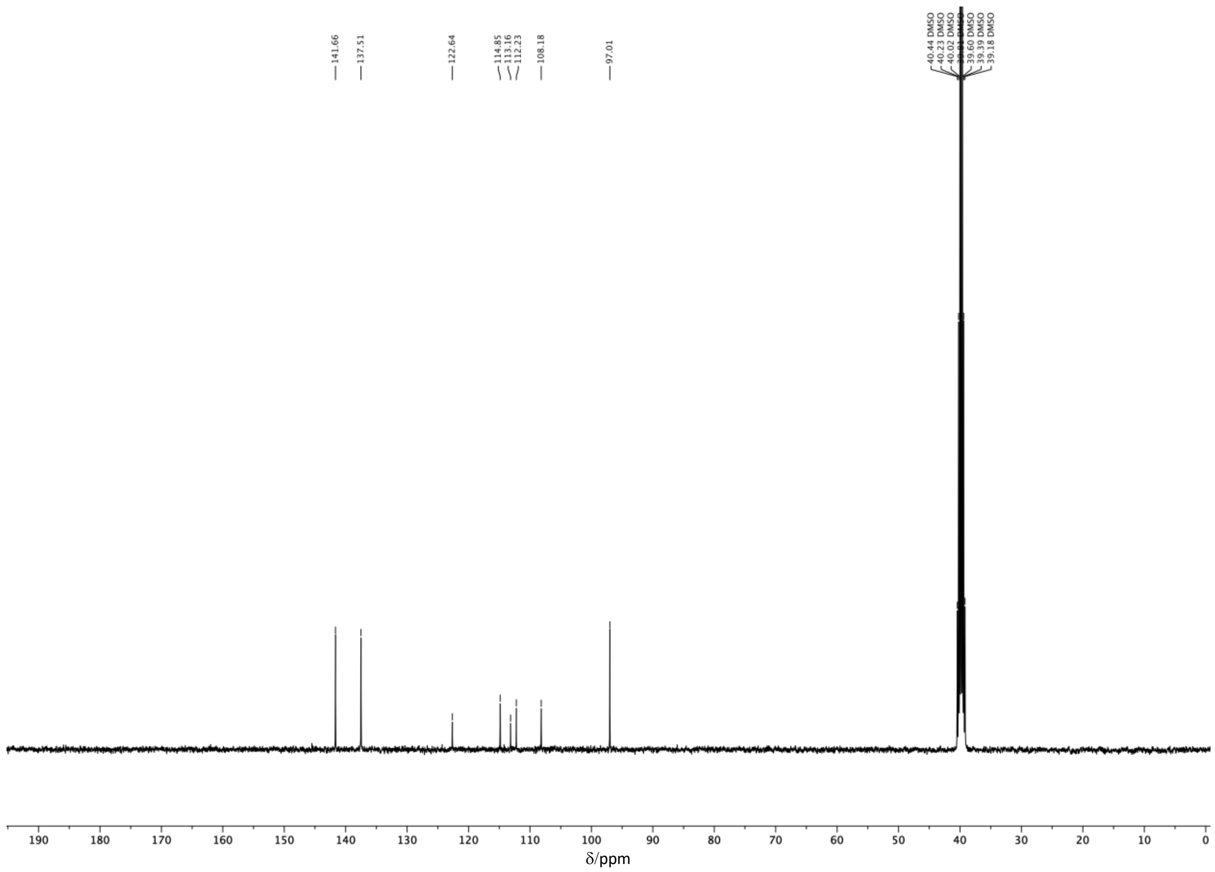

Figure S3 ^{13}C NMR spectrum of cyanovinyl 4,5-dicyanoimidazole **20** obtained following the preparation of cyanovinyl 4,5-dicyanoimidazole **20** (see General Procedures). (DMSO-d_6) δ 141.7, 137.5, 122.6, 114.9, 113.2, 112.2, 108.2, 97.0.

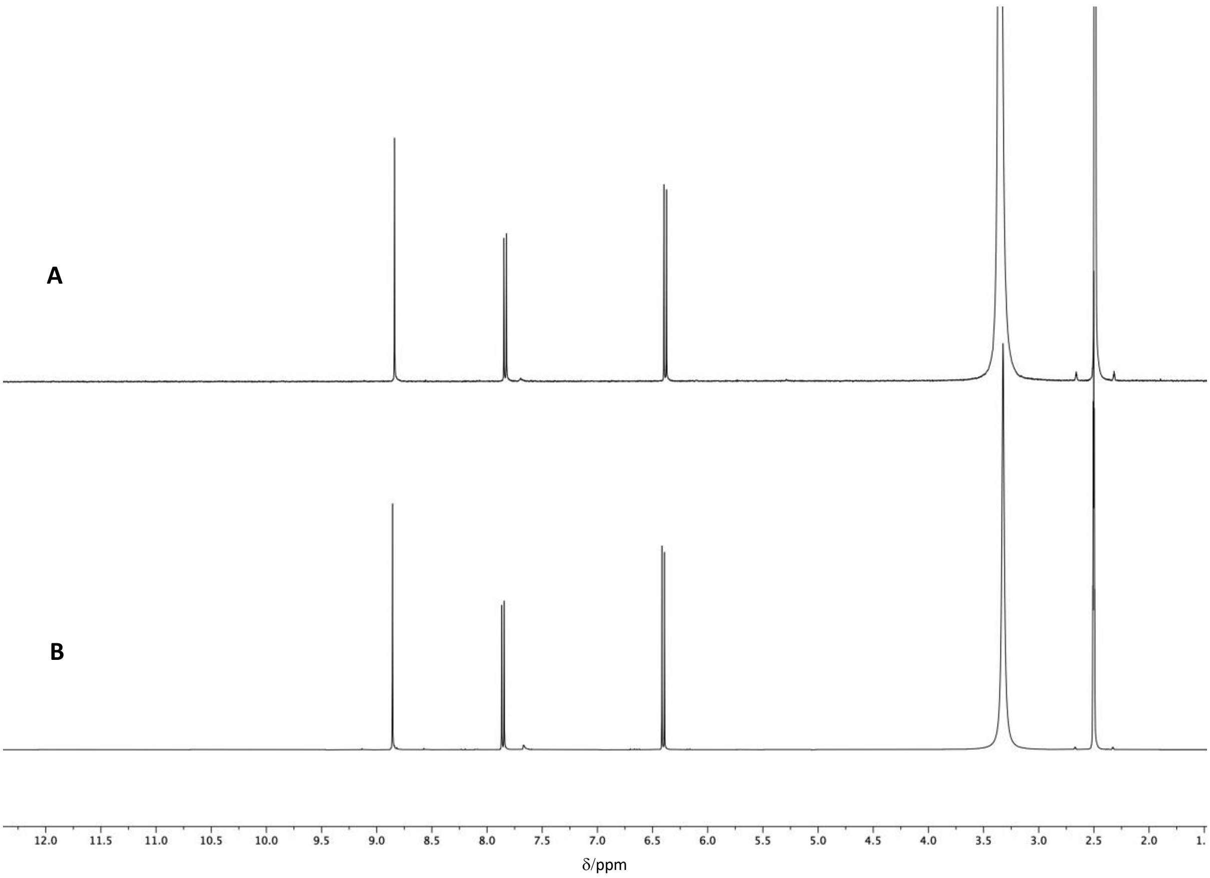

Figure S4 Stability of CV-DCI **20** under an oxic atmosphere. A – ^{1}H NMR Spectrum of a sample of **20** (as prepared in General Procedures) in DMSO-d_6; B – A portion of **20**, as prepared for spectrum A, was stored in solid form in a vial on the bench for 6 months, then spectrum B acquired in DMSO-d_6.

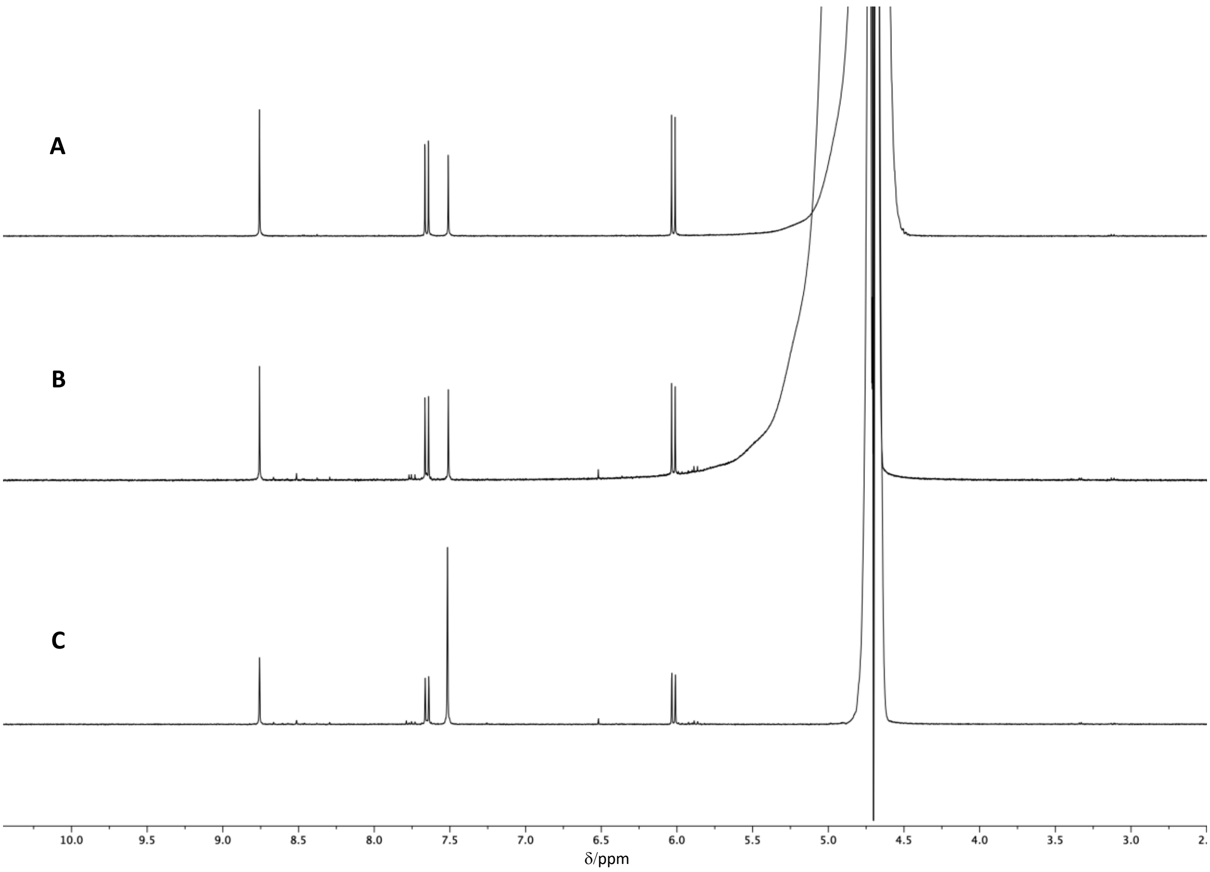

Figure S5 Stability of CV-DCI **20** in H_2O in the presence of cyanide and orthophosphate. To a solution of KCN (3 mg, 0.050 mmol) and $NaH_2PO4.2H_2O$ (8 mg, 0.050 mmol) in H_2O/D_2O (9:1, 1 mL) at pH 6.5, was added **20** (8 mg, 0.050 mmol), and the mixture gently tumbled. A – An aliquot of the solution removed after 2 h, diluted with D_2O, and this ^{1}H NMR spectrum was acquired; B – An aliquot of the solution removed after 4 d, diluted with D_2O, and this ^{1}H NMR spectrum was acquired; C – As spectrum B, spiked with 4,5-dicyanoimidazole **18**.

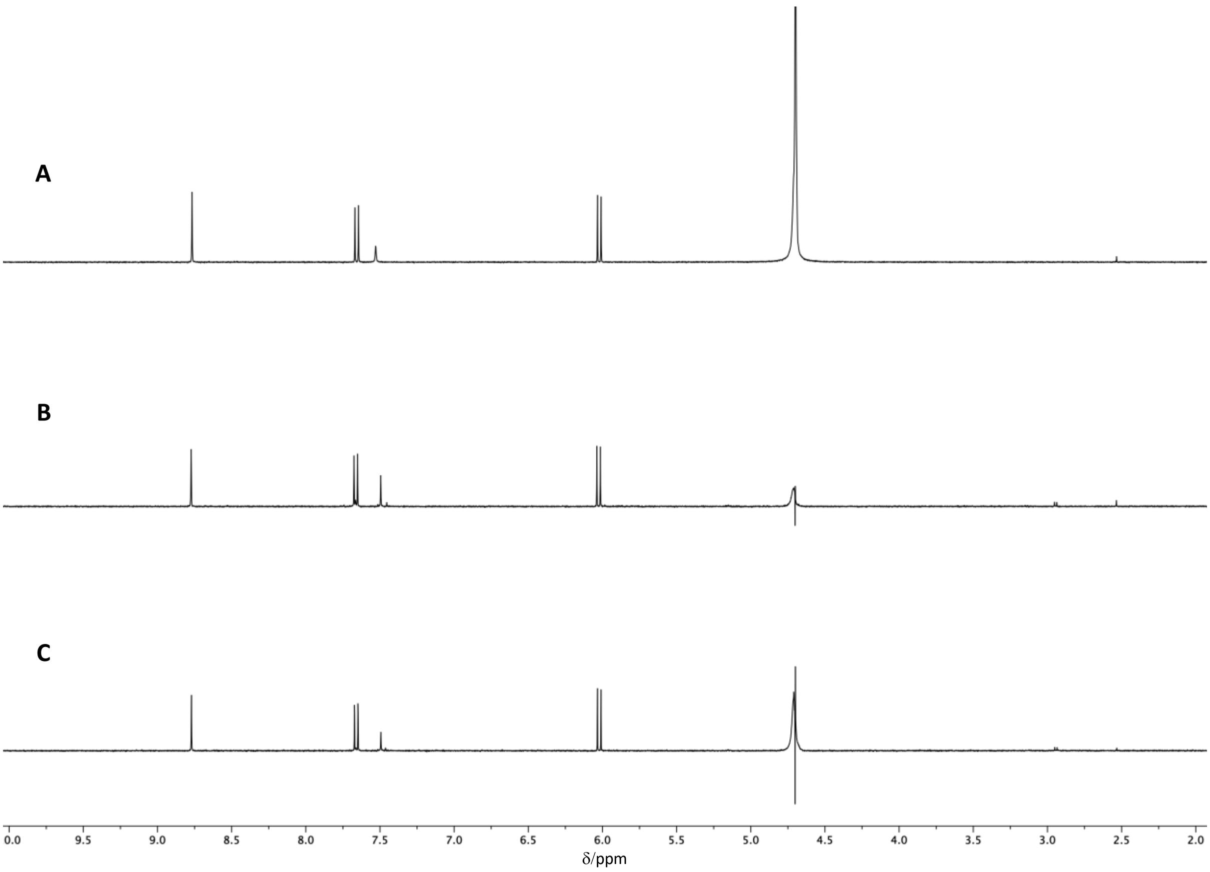

Figure S6 Stability of CV-DCI **20** in H_2O in the presence of ammonia. To a solution of NH_4Cl (8 mg, 0.150 mmol) in H_2O/D_2O (9:1, 1 mL) at pH 7.1 was added **20** (8 mg, 0.050 mmol), and the mixture gently tumbled. After 2 d an aliquot of the solution was removed, diluted with D_2O and spectrum A acquired. Further NH_4Cl (10 mg, 0.187 mmol) was added and the pH increased to 8.5. After 24 h, an aliquot of the solution was removed, diluted with D_2O, and spectrum B acquired. After a further 2 d, an aliquot of the solution was removed, diluted with D_2O, and spectrum C acquired.

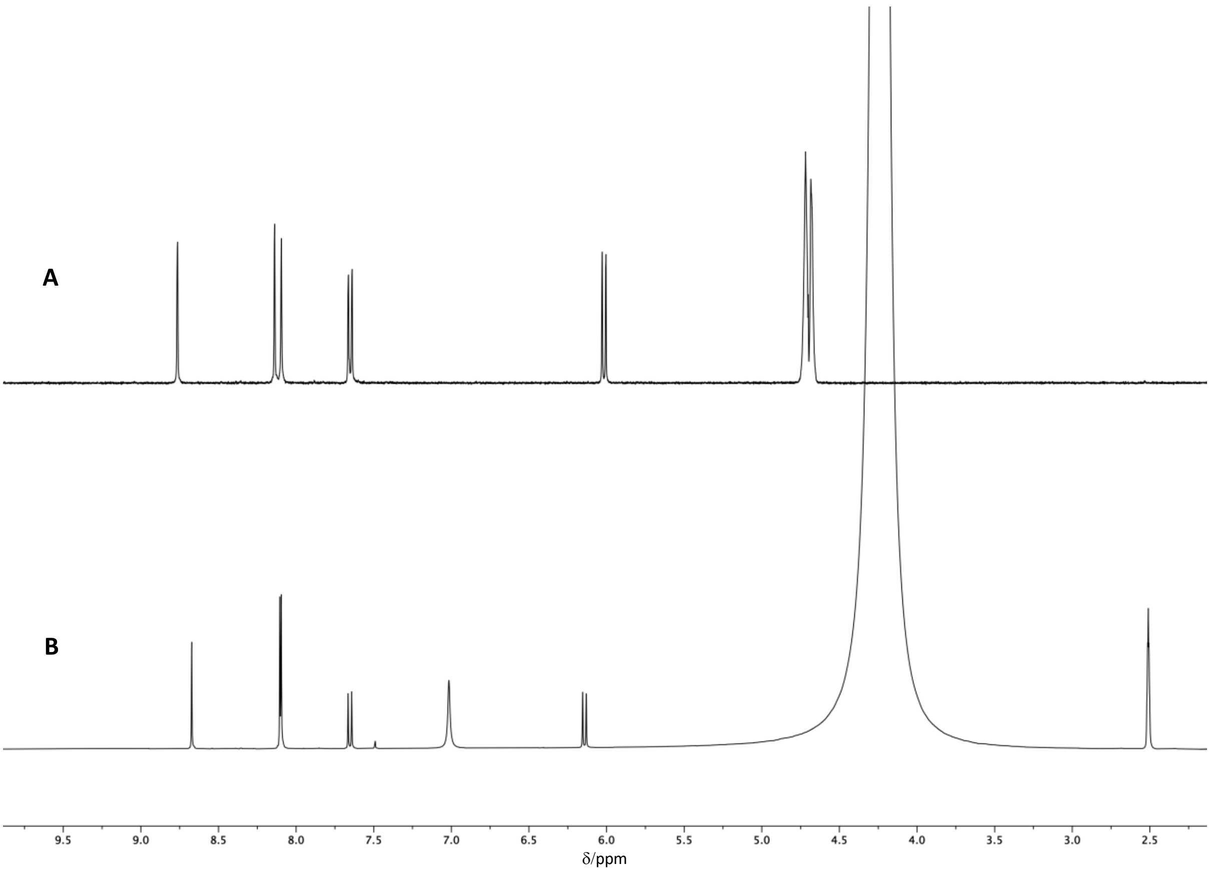

Figure S7 Stability of CV-DCI **20** in H_2O in the presence of adenine **17**. Adenine **17** (7 mg, 0.050 mmol) and **20** (8 mg, 0.050 mmol) were suspended in H_2O/D_2O (9:1, 0.5 mL) and the mixture was heated to 45 °C with stirring. After 4 h an aliquot of the solution was removed, diluted with D_2O and spectrum A acquired. After 4 d, the reaction was centrifuged and the supernatant discarded. The solid residue was dissolved in DMSO-d_6 and spectrum B acquired.

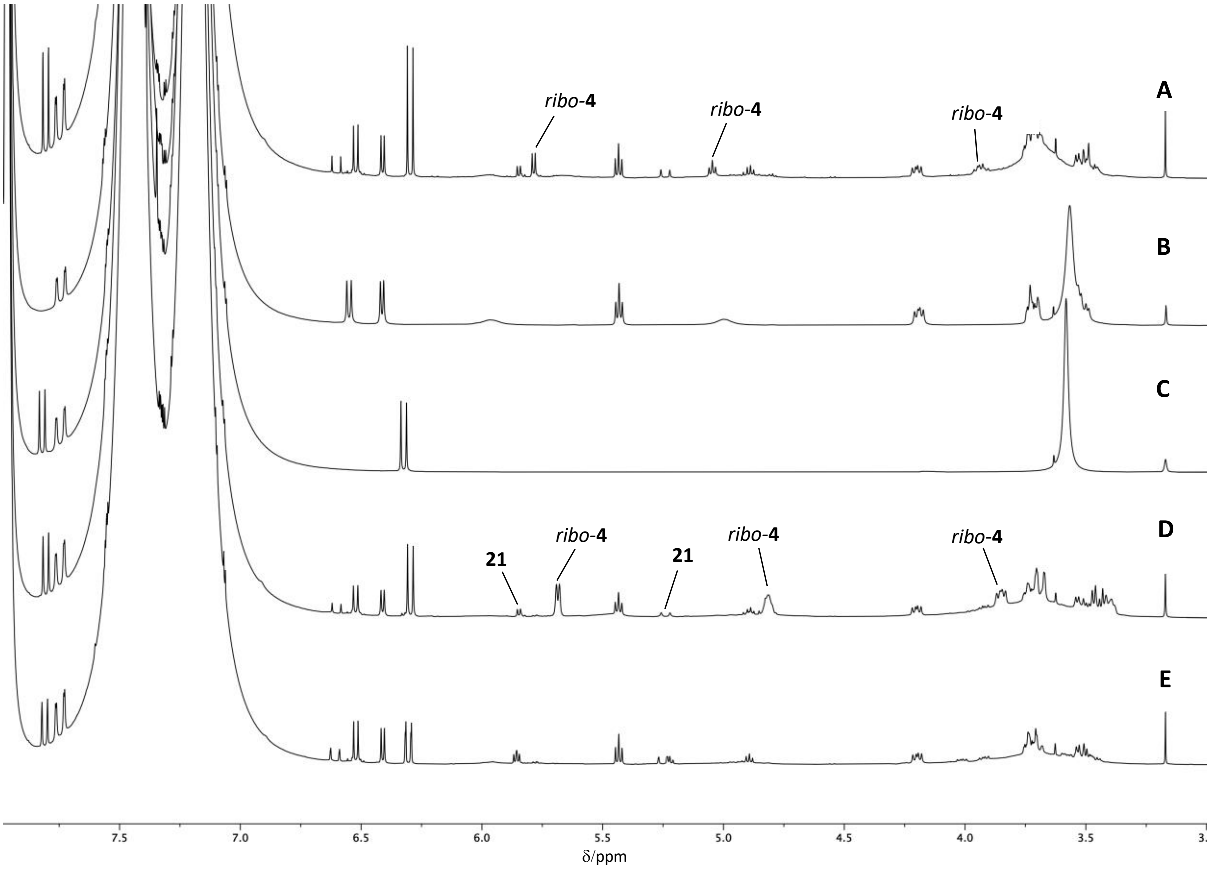

Figure S8 Reaction of *ribo*-aminooxazole *ribo*-**4** with CV-DCI **20** in formamide. *Ribo*-**4** (9 mg, 0.050 mmol) and **20** (17 mg, 0.100 mmol) were added to formamide (0.5 mL), the reaction sealed and then heated to 45 °C with stirring. At the desired timepoint, a portion (90 μL) was diluted into DMSO-d_6, an aliquot of a standard (NaOAc in formamide (100 mM) 10 μL) added and a ^{1}H NMR spectrum acquired. A – ^{1}H NMR Spectrum of the reaction after 20 h; B – ^{1}H NMR Spectrum of an authentic sample of *ribo*-anhydrocytidine **6** in formamide/DMSO-d_6 (1:4); C – ^{1}H NMR Spectrum of an authentic sample of **20** in formamide/DMSO-d_6 (1:4. Note: the corresponding doublet is seen at 7.81 ppm); D – As spectrum A, spiked with an authentic sample of *ribo*-**4**. The signals have shifted slightly relative to spectrum A, which is presumably due to the basic nature of the neutral form of *ribo*-**4**. The compound denoted **21** is assumed to be *trans*-cyanovinylated *ribo*-aminooxazole which cannot undergo cyclisation to **6** (one doublet of the cyanovinyl group can be seen at 5.23 ppm, *J* = 14.7 and its coupling partner can be seen in Figure S9); E – ^{1}H NMR Spectrum of the reaction after 44 h. The signal at 3.33 ppm is MeOH impurity in the formamide

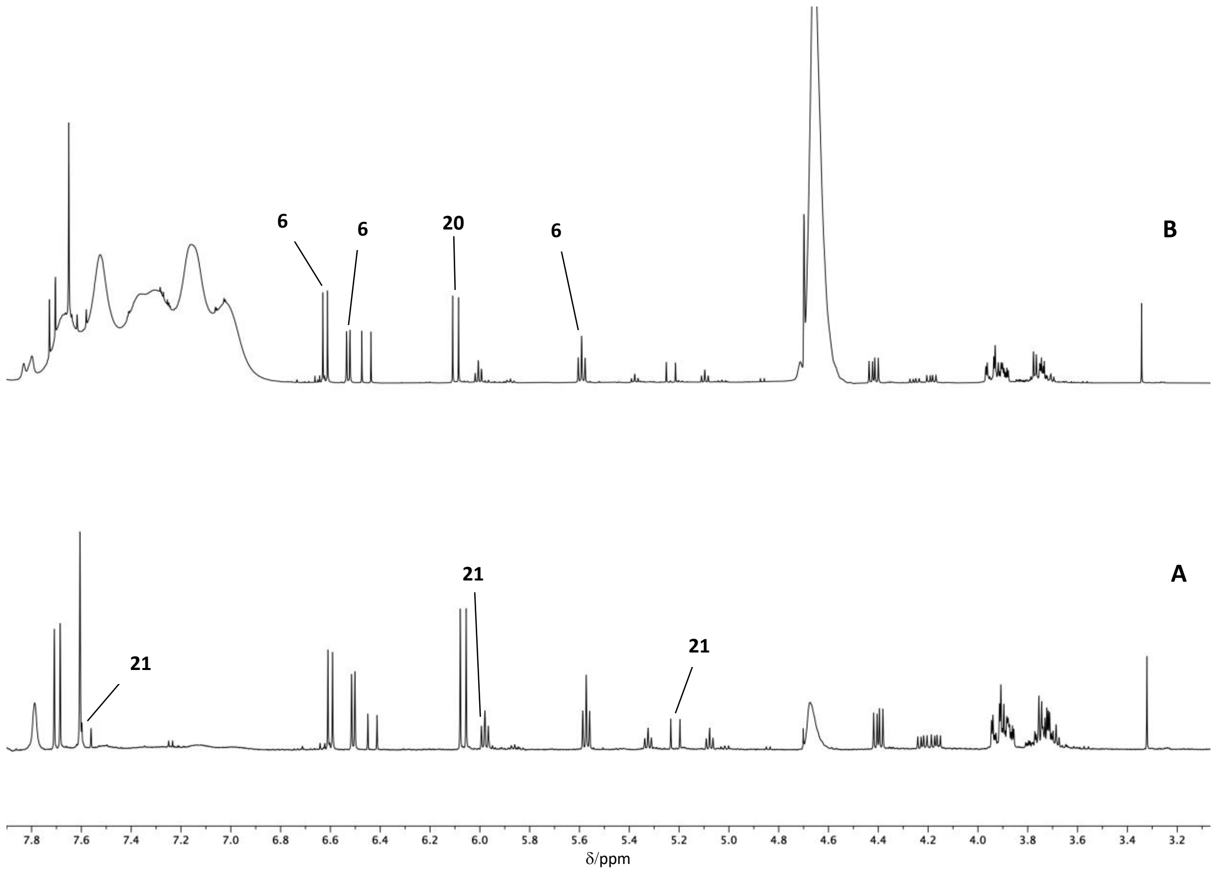

Figure S9 Reaction of *ribo*-aminooxazole *ribo*-**4** with CV-DCI **20** in formamide. A – As Figure S8, spectrum A, but the reaction was run for 24 h and then a portion (90 μL) diluted into H_2O/D_2O with an aliquot of a standard (NaOAc in formamide (100 mM) 10 μL). The solvent suppression NMR experiment run in spectrum A, for some reason, also suppressed the formamide signals (see spectrum B, 6.8-7.8 ppm), but this allowed the two vinyl signals of *trans*-cyanovinylated *ribo*-aminooxazole **21** to be seen (7.58 ppm (d, J = 14.7) and 5.22 ppm (d, J = 14.7)); B – As spectrum A, but after 2 d reaction. The signal at 5.9 ppm is actually two overlapped doublets, one belonging to **21** and the other to *ribo*-**4**, the doublet at 6.42 ppm is thought to be *trans*-cyanovinyl 4,5-dicyanoimidazole and the signal at 3.33 ppm is MeOH impurity in the formamide. **6** = *ribo*-Anhydrocytidine.

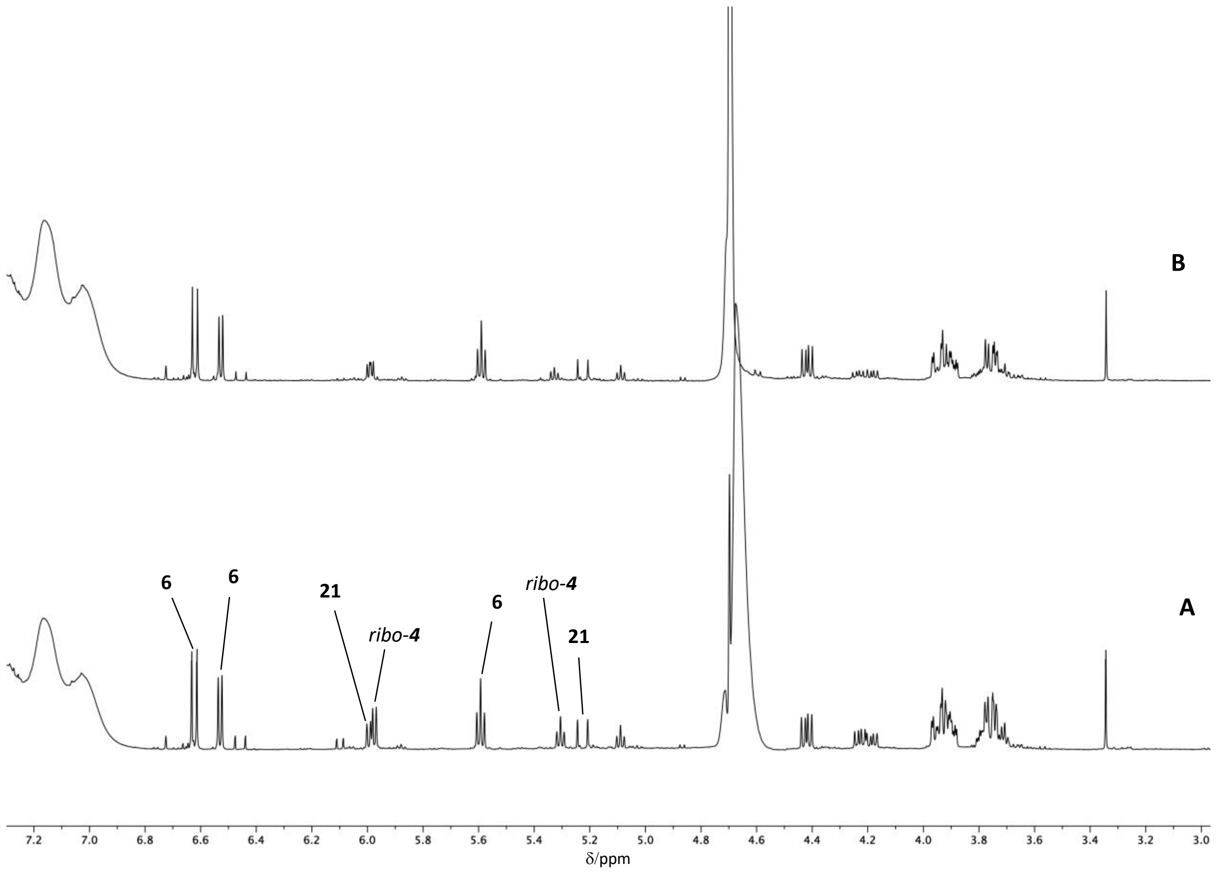

Figure S10 Reaction of *ribo*-aminooxazole *ribo*-**4** with CV-DCI **20** in formamide and thermal stability of *trans*-cyanovinylated *ribo*-aminooxazole **21**. *Ribo*-**4** (9 mg, 0.050 mmol) and **20** (8 mg, 0.050 mmol) were added to formamide (0.5 mL), the reaction sealed and then heated to 45 °C with stirring. After 3 d, a portion (90 μL) was diluted into H_2O/D_2O and an aliquot of a standard (NaOAc in formamide (100 mM) 10 μL) added, then a ^{1}H NMR spectrum (spectrum A) was acquired. The remaining formamide solution was then heated to 90 C for 3.5 h, then cooled to RT and a portion (90 μL) was diluted into H_2O/D_2O. An aliquot of a standard (NaOAc in formamide (100 mM) 10 μL) added, then a ^{1}H NMR spectrum (spectrum B) was acquired. In spectrum A, there is ~ 40% of *ribo*-anhydrocytidine **6**, ~ 14% of *trans*-cyanovinylated *ribo*-aminooxazole **21** and ~ 22% of *ribo*-**4**. In spectrum B, there is ~ 42% of *ribo*-anhydrocytidine **6**, ~ 14% of *trans*-cyanovinylated *ribo*-aminooxazole **21** and ~ 14% of *ribo*-**4**.

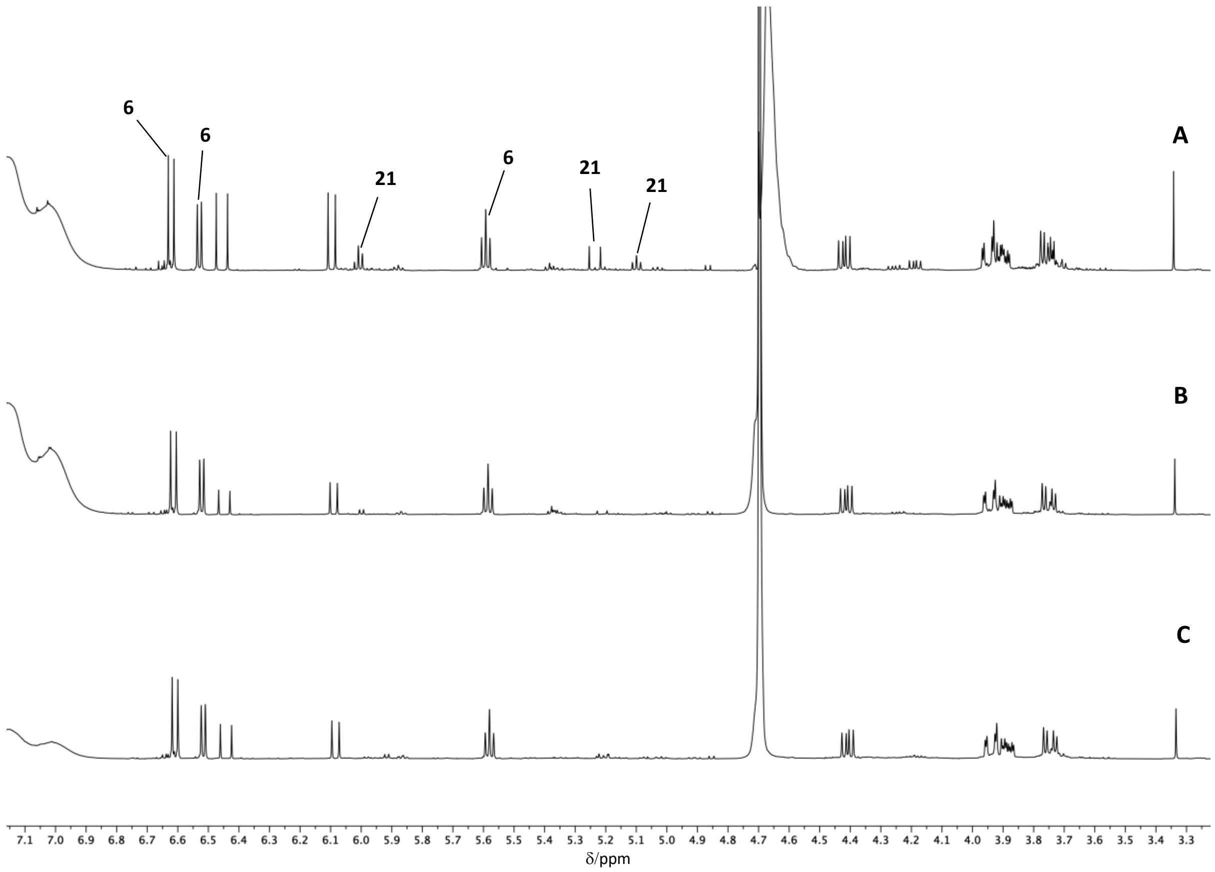

Figure S11 Reaction of *ribo*-aminooxazole *ribo*-**4** with CV-DCI **20** in formamide followed by UV irradiation. *Ribo*-**4** (9 mg, 0.050 mmol) and **20** (17 mg, 0.100 mmol) were added to formamide (0.5 mL), the reaction sealed and then heated to 45 °C with stirring. After 3 d, a portion (90 μL) was diluted into H_2O/D_2O and an aliquot of a standard (NaOAc in formamide (100 mM) 10 μL) added, then a ^{1}H NMR spectrum (spectrum A) was acquired using a quartz NMR spectroscopy tube; B – As spectrum A, after the NMR tube containing the sample had been subjected to UV irradiation for 2 h; C – A sample (90 μL) of the pure formamide reaction was transferred to a quartz cuvette and subjected to UV irradiation for 3.5 h. This solution was then diluted with H_2O/D_2O and an aliquot of a standard (NaOAc in formamide (100 mM) 10 μL) added, and a ^{1}H NMR spectrum (spectrum C) acquired. In spectrum A, there is ~ 48% of *ribo*-anhydrocytidine **6**, ~ 14% of *trans*-cyanovinylated *ribo*-aminooxazole **21** and ~ 7% of *ribo*-**4**. In spectrum B, there is ~ 58% of *ribo*-anhydrocytidine **6**, ~ 7% of *ribo*-**4** and **21** has been consumed. In spectrum C, there is ~ 55% of *ribo*-anhydrocytidine **6**, ~ 7% of *ribo*-**4** and **21** has been consumed.

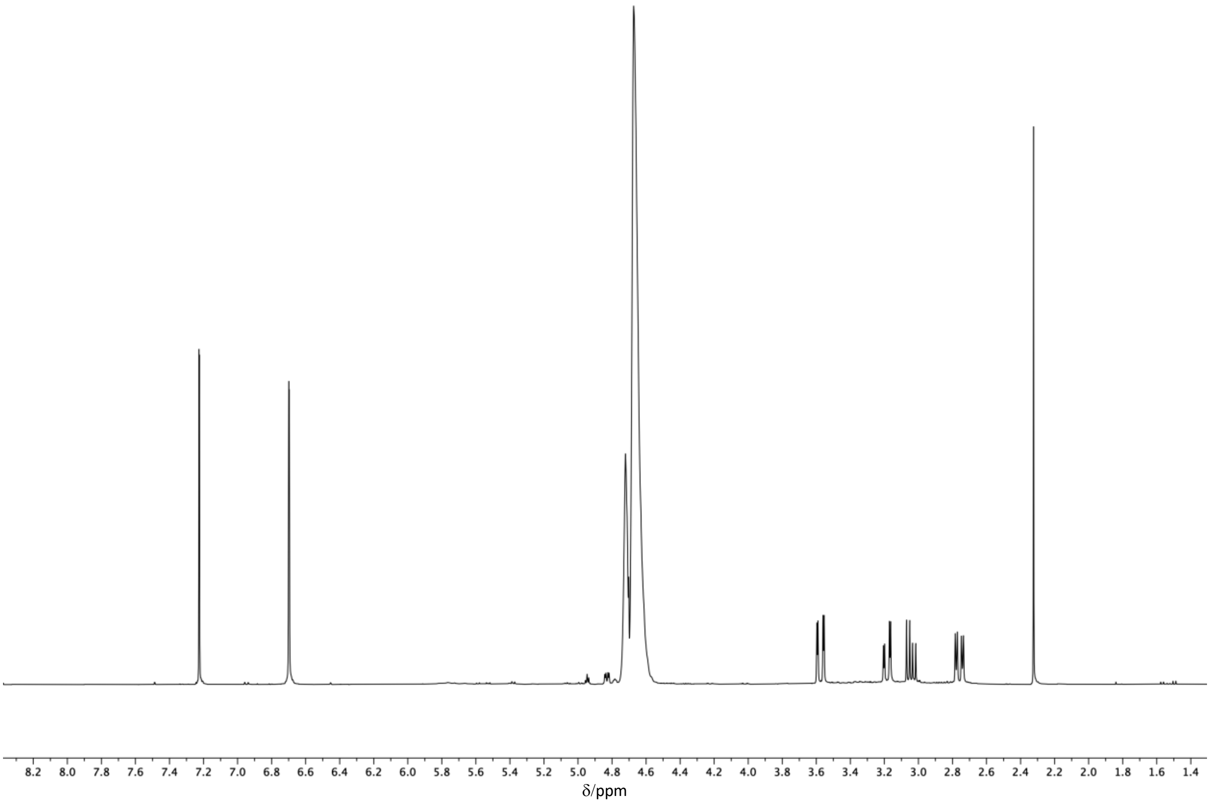

Figure S12 Attempted synthesis of 2-aminothiazole **23** from 2-aminooxazole **2**. To an Eppendorf tube was charged NaSH.xH_2O (> 60%, 9 mg, 0.100 mmol), degassed 10% D_2O in H_2O (0.6 mL) and Na_2HPO_4 (29 mg, 0.200 mmol). The pH was adjusted to 7 and the volume made up to 1 mL, then **2** (8 mg, 0.100 mmol) was added, the vessel sealed and heated to 40 °C for 24 h then the above ^{1}H NMR spectrum acquired. Singlet at 2.3 ppm is succinate, added as a standard.

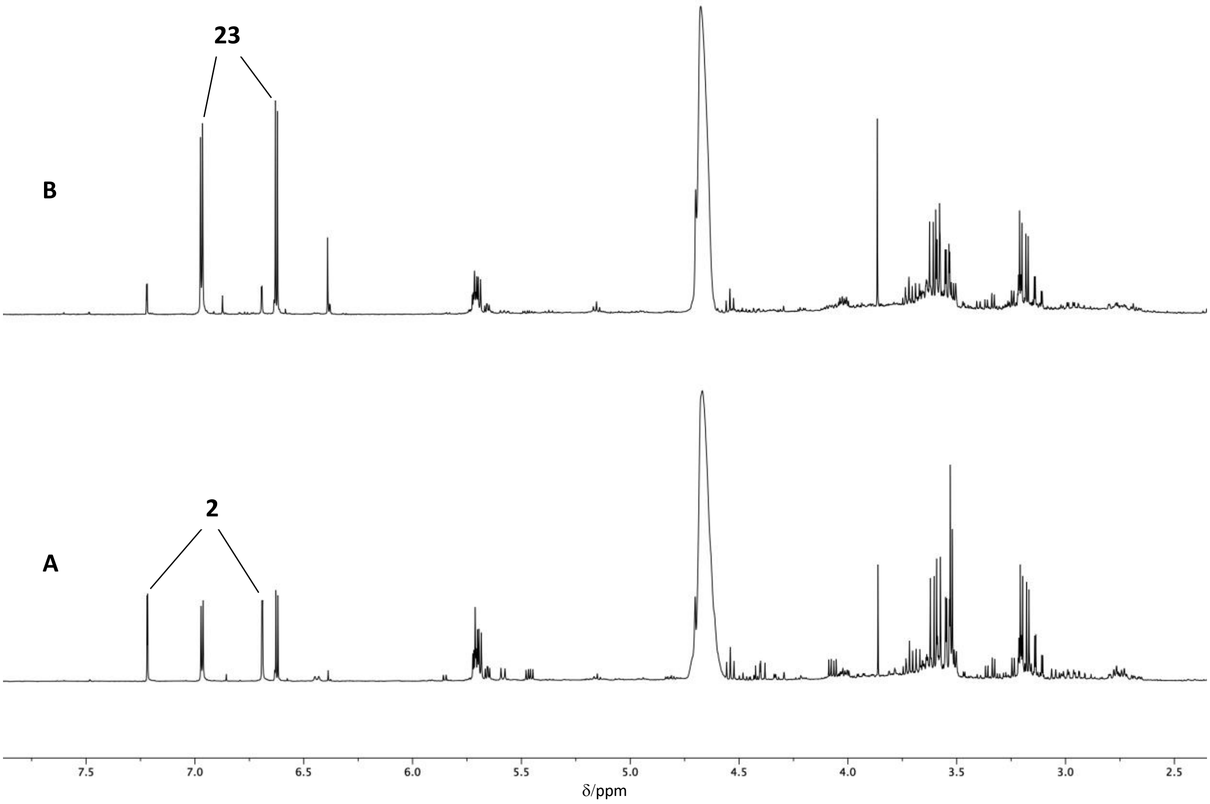

Figure S13 Synthesis of 2-aminothiazole **23** from glycolaldehyde **1** at higher concentration in phosphate buffer. To an Eppendorf tube was charged NaSH.xH_2O (> 60%, 28 mg, 0.300 mmol), degassed 10% D_2O in H_2O (1 mL) and Na_2HPO_4 (57 mg, 0.400 mmol). The pH was adjusted to 7.0 and the volume made up to 2 mL, then **1** (12 mg, 0.200 mmol) and NH_2CN (13 mg, 0.300 mmol) were added. The vessel was sealed and heated to 60 °C for the desired amount of time, then a portion (450 μL) was removed, succinate added as a standard for integration (not shown) and a ^{1}H NMR spectrum acquired. A – ^{1}H NMR Spectrum of the crude reaction mixture after 24 h; B – ^{1}H NMR Spectrum of the crude reaction mixture after 5 d.

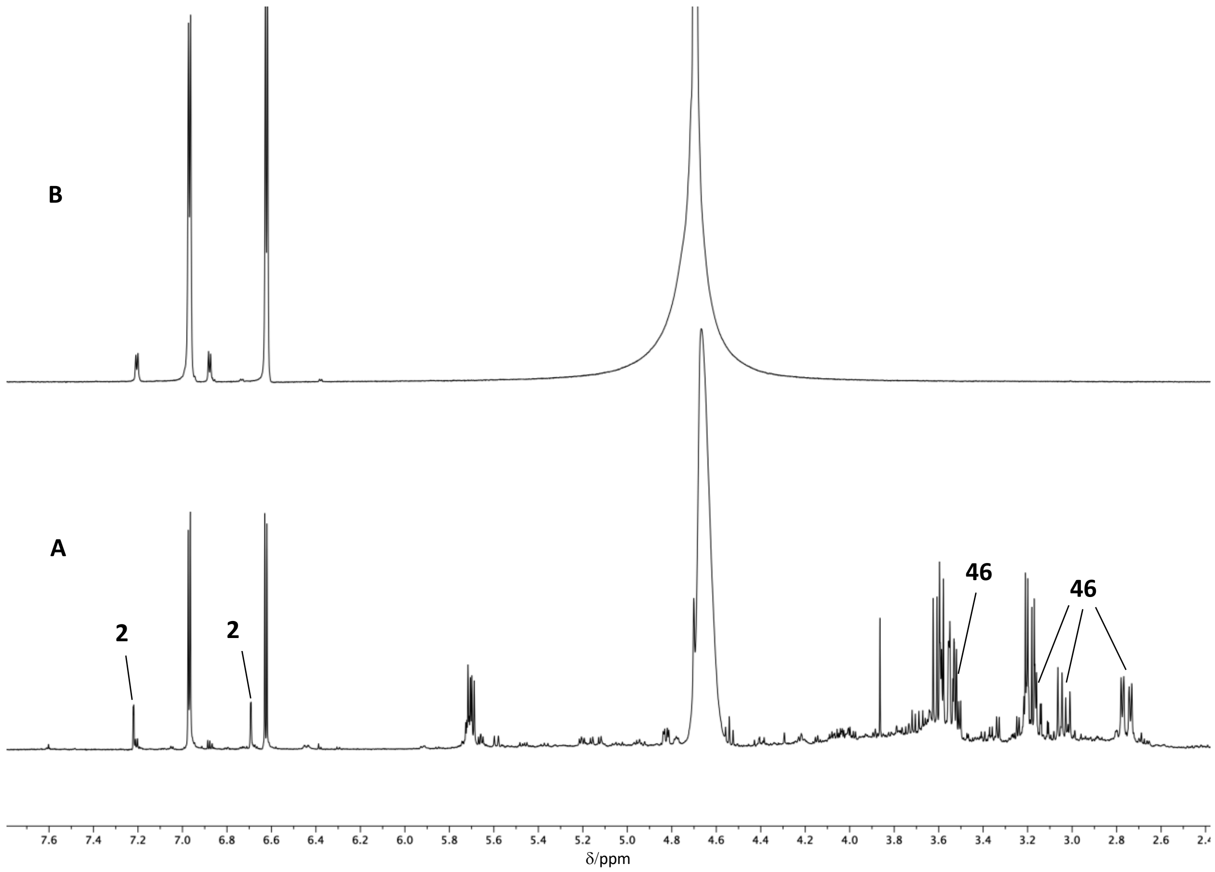

Figure S14 Synthesis of 2-aminothiazole **23** from glycolaldehyde **1** at higher concentration in bicarbonate buffer. To an Eppendorf tube was charged $NaSH.xH_2O$ (> 60%, 28 mg, 0.300 mmol), degassed 10% D_2O in H_2O (1 mL) and $NaHCO_3$ (34 mg, 0.400 mmol). The pH was adjusted to 9.2 and the volume made up to 2 mL, then **1** (12 mg, 0.200 mmol) and NH_2CN (13 mg, 0.300 mmol) were added. The vessel was sealed and heated to 60 °C for the desired amount of time, then a portion (450 μL) was removed, succinate added as a standard for integration (not shown) and a ^1H NMR spectrum acquired. A – ^1H NMR Spectrum of the crude reaction mixture after 24 h; B – ^1H NMR Spectrum of a commercial sample of **23** in bicarbonate buffer at pH 9.2 after 2 h (note the downfield pair of doublets (δ = 7.21 and 6.88 ppm, J = 3.7) which are ascribed to the CO_2 adduct of **23** and also observed in the reaction mixture (spectrum A)).

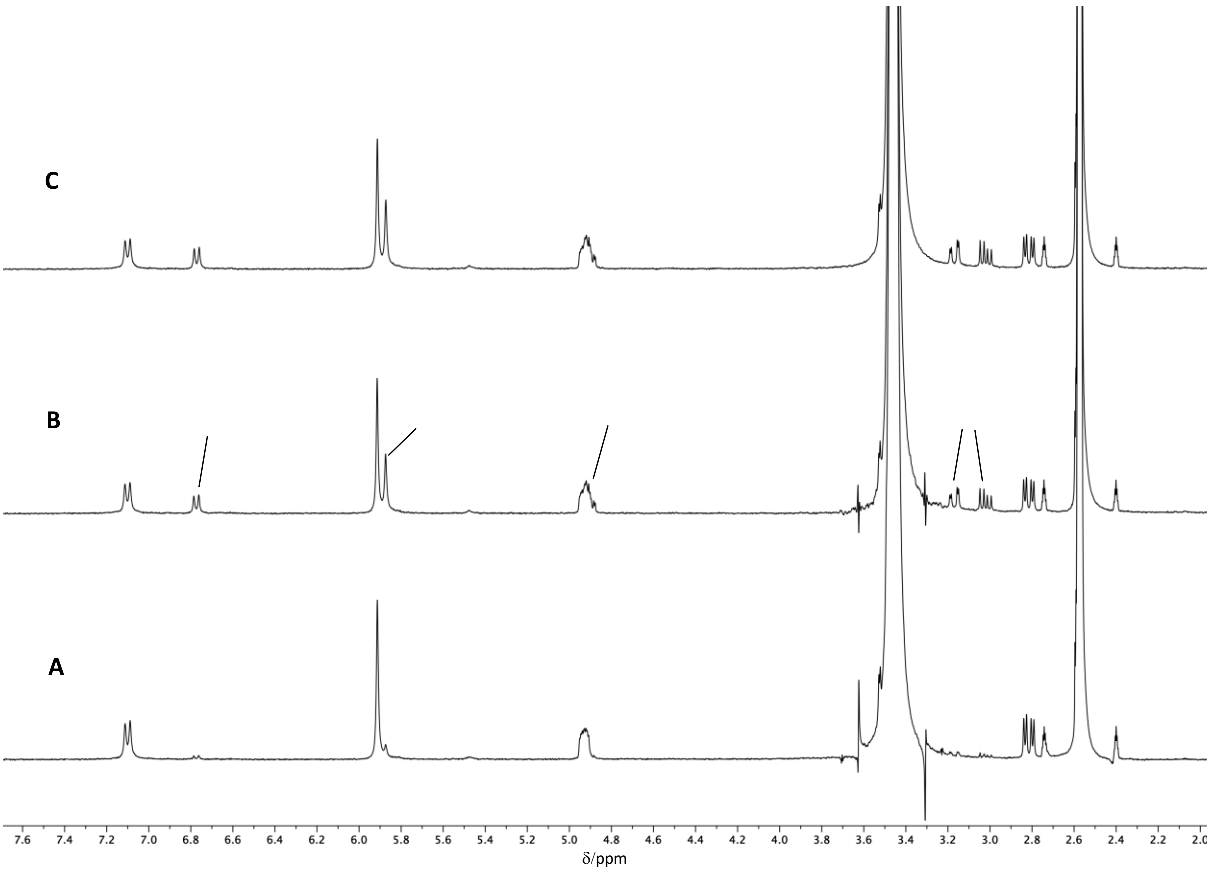

Figure S15 Equilibration of *trans*-**46**. To an Eppendorf tube was charged NaSH.xH_2O (> 60%, 14 mg, 0.150 mmol), degassed 10% D_2O in H_2O (0.6 mL) and $NaHCO_3$ (17 mg, 0.200 mmol). The pH was adjusted to 7 and the volume made up to 1 mL, then glycolaldehyde **1** (6 mg, 0.100 mmol) and NH_2CN (6 mg, 0.150 mmol) were added, and the vessel sealed and heated to 60 °C for 24 h. The reaction was cooled to RT, the mother liquors removed using a glass pipette and the majority of the remaining liquid removed with a paper towel. DMSO-d_6 (0.6 mL) Was added and the suspension sonicated for 30 s. The liquid was transferred to an NMR tube and a ^{1}H spectrum (spectrum A) recorded; B – As spectrum A, after 8 h in DMSO-d_6; C – As spectrum A, after 17 h in DMSO-d_6. Ratio of diastereomers = 94:6 in spectrum A, 70:30 in spectrum B, 66:34 in spectrum C.

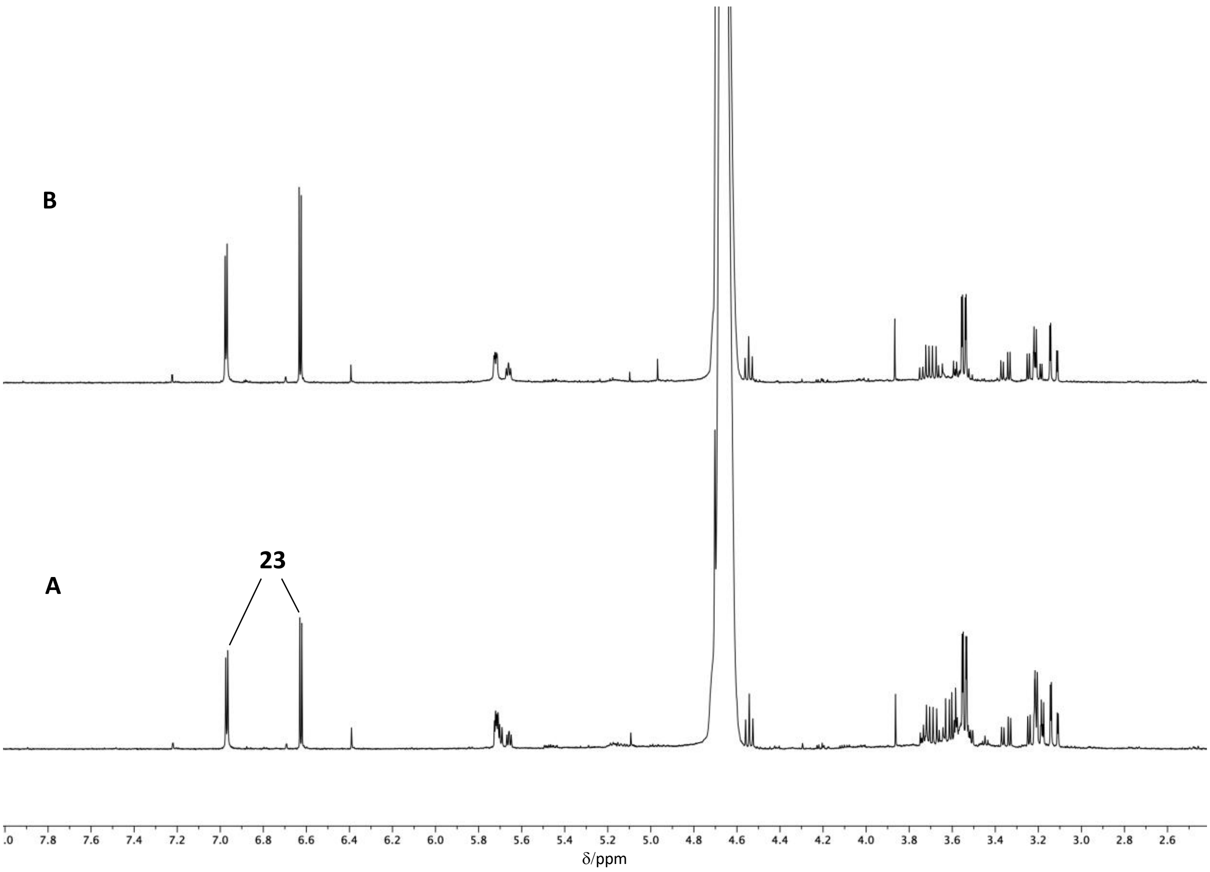

Figure S16 Synthesis of 2-aminothiazole **23** from glycolaldehyde **1** at lower concentration in phosphate buffer. To an Eppendorf tube was charged NaSH.xH_2O (> 60%, 19 mg, 0.200 mmol), degassed 10% D_2O in H_2O (1 mL) and Na_2HPO_4 (57 mg, 0.400 mmol). The pH was adjusted to 7.0 and the volume made up to 2 mL, then **1** (3 mg, 0.050 mmol) and NH_2CN (6 mg, 0.150 mmol) were added. The vessel was sealed and heated to 60 °C for the desired amount of time, then a portion (450 μL) was removed, succinate added as a standard for integration (not shown) and a ^{1}H NMR spectrum acquired. A – ^{1}H NMR Spectrum of the crude reaction mixture after 3 d; B – ^{1}H NMR Spectrum of the crude reaction mixture after 7 d.

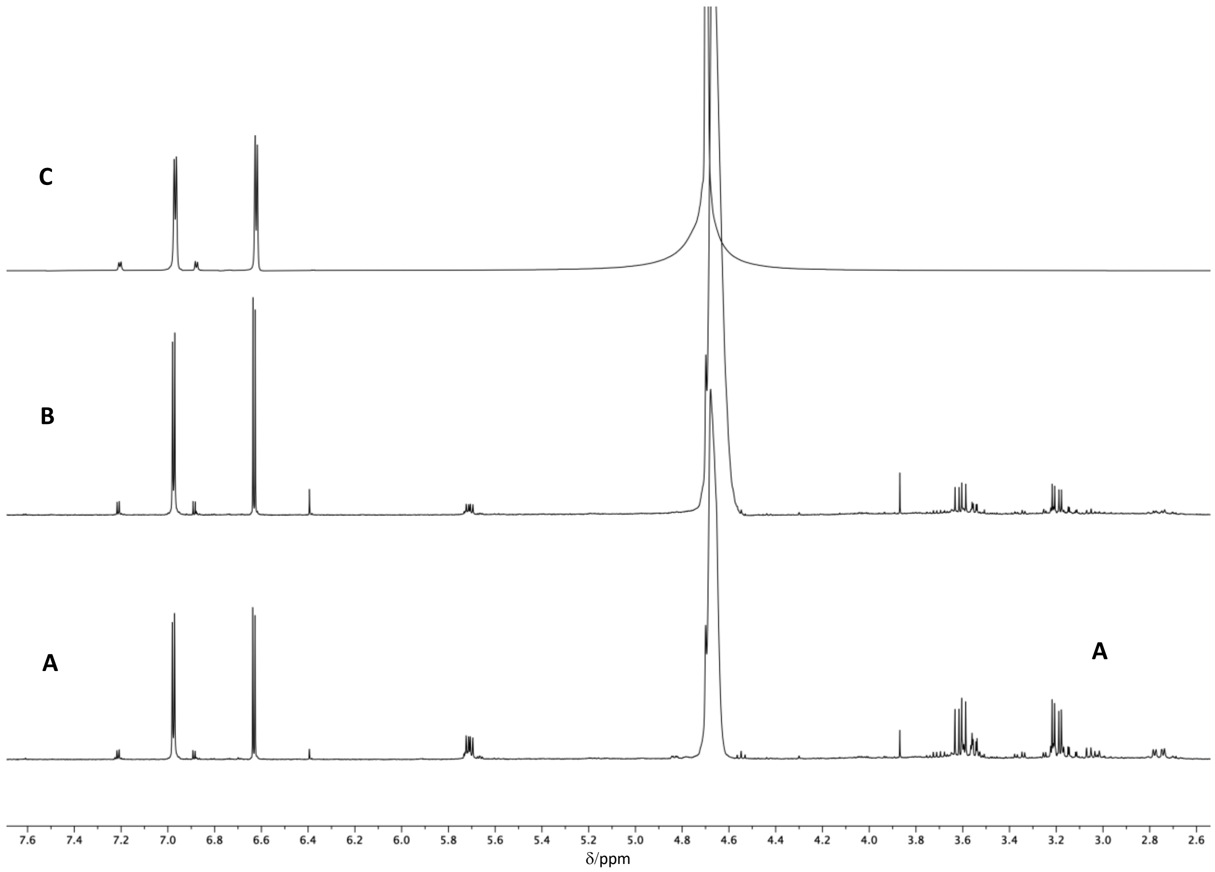

Figure S17 Synthesis of 2-aminothiazole **23** from glycolaldehyde **1** at lower concentration in bicarbonate buffer. To an Eppendorf tube was charged $NaSH.xH_2O$ (> 60%, 19 mg, 0.200 mmol), degassed 10% D_2O in H_2O (1 mL) and $NaHCO_3$ (34 mg, 0.400 mmol). The pH was adjusted to 9.2 and the volume made up to 2 mL, then **1** (3 mg, 0.050 mmol) and NH_2CN (6 mg, 0.150 mmol) were added. The vessel was sealed and heated to 60 °C for the desired amount of time, then a portion (450 μL) was removed, succinate added as a standard for integration (not shown) and a ^1H NMR spectrum acquired. A – ^1H NMR Spectrum of the crude reaction mixture after 3 d; B – ^1H NMR Spectrum of the crude reaction mixture after 7 d; C – ^1H NMR Spectrum of **23** in bicarbonate buffer at pH 9.3 after 2 h (note the downfield pair of doublets (δ = 7.21 and 6.88 ppm, J = 3.7) which are ascribed to the CO_2 adduct of **23** and also observed in the reaction mixture (spectrum A)).

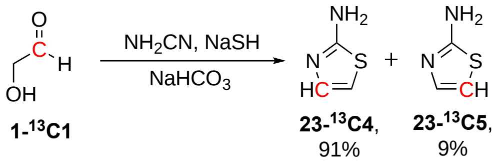

Figure S18. Positioning of labelled and unlabelled carbons in **23** when derived from ^{13}C-labelled **1**. Red atoms denote ^{13}C-labelling. The conversion of glycolaldehyde **1** to mercaptoacetaldehyde **24** *via* the thiocarbonyl analogue of **1** seemed highly unlikely given the usual requirement for specific acid catalysis for such a process. Nonetheless, we employed glycolaldehyde-1-^{13}C **1-^{13}C1** in the optimized reaction to follow the position of labelled and unlabelled carbon atoms in **23** when derived from **1**, NaSH and NH_2CN in bicarbonate buffer. In thus-formed 2-aminothiazole **23**, we observed a product distribution of 91:9 of isotopomers, and the major product was assigned as having the labelled carbon atom (C1 derived from **1**) attached to nitrogen in **23** *i.e.* **23-^{13}C4** (Figures S18 and S19). This assignment was based on the coupling constants, chemical shifts of ^{1}H and ^{13}C NMR signals and by comparison of ^{13}C chemical shifts to commercially available 2-amino-5-methylthiazole (Figures S19-S21). Whilst the presence of **23-^{13}C5** could be explained by the *in situ* formation of a small amount of **24**, the fact that isomerization of **1-^{13}C1** to glycolaldehyde-2-^{13}C **1-^{13}C2** occurs readily in bicarbonate buffer at 60 °C (~ 33% after 15 min reaction) provides a more likely explanation.

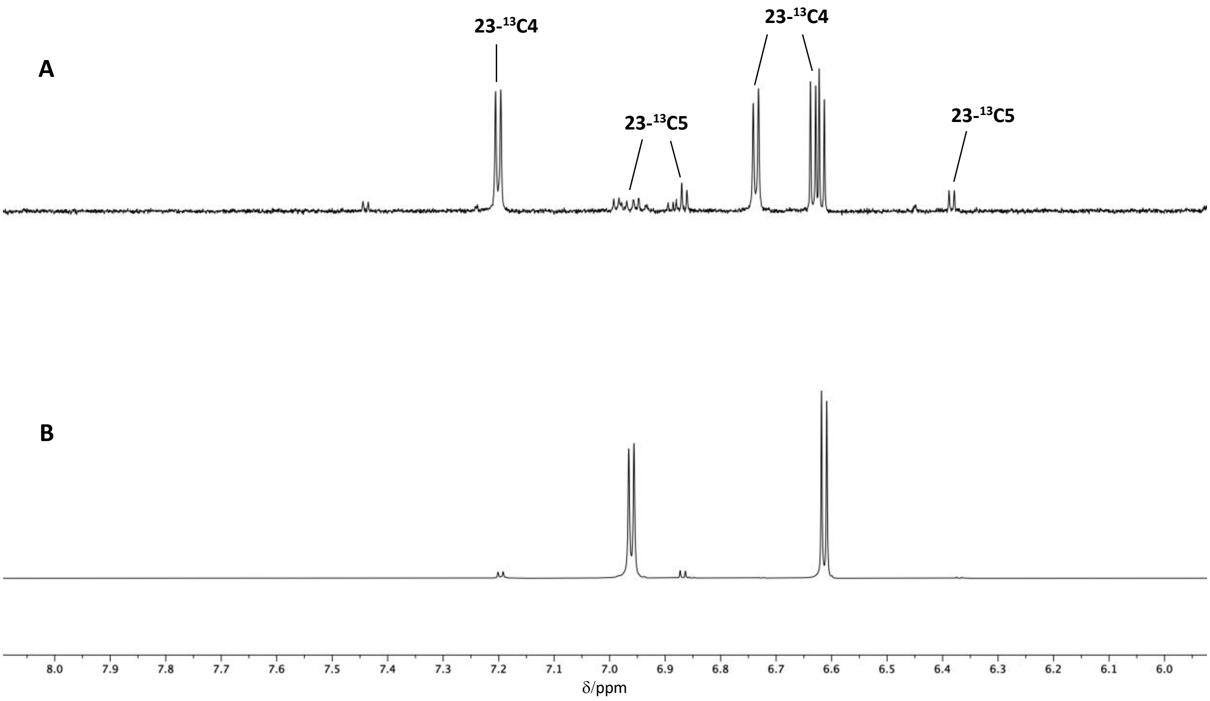

Figure S19 Synthesis of 2-aminothiazole **23** using glycolaldehyde-1-^{13}C **1-^{13}C1**. A – $NaHCO_3$ (34 mg, 0.400 mmol) and NaSH (19 mg, 0.200 mmol) were dissolved in degassed D_2O/H_2O (1:9, 1 mL) and the pH adjusted to 9.1. A solution of **1-^{13}C1** in H_2O (~ 240mM, 210 μL) was added followed immediately by NH_2CN (6 mg, 0.150 mmol) and the volume made up to 2 mL with degassed D_2O/H_2O (1:9). The Eppendorf tube was sealed and heated to 60 °C for 28 h, after which time a ^{1}H NMR spectrum (spectrum A) was acquired; B – A ^{1}H NMR spectrum of a commercial sample of **23** in bicarbonate buffer at pH 9.2 [δ 6.96 (d, *J* = 3.9), 6.61 (d, *J* = 3.9)]. The pair of doublets at 7.12 ppm and 6.87 ppm (*J* = 3.7) are presumed to belong to the cabamic acid anion resulting from addition of **23** to CO_2. By comparison to spectrum B, the downfield proton signal has been split by a typically large one-bond ^{13}C-^{1}H coupling ($^1J_{CH}$ = 186), whereas the upfield proton has a ^{13}C-^{1}H coupling consistent with a geminal coupling ($^2J_{CH}$ = 6.3). The small amount of the other isotopomer can also be seen: δ 6.96 (dd, *J* = 8.5, 3.8, 1 H), 6.63 (dd, *J* = 193, 3.8, 1 H). The ^{13}C NMR spectra were also compared, see Figure S20.

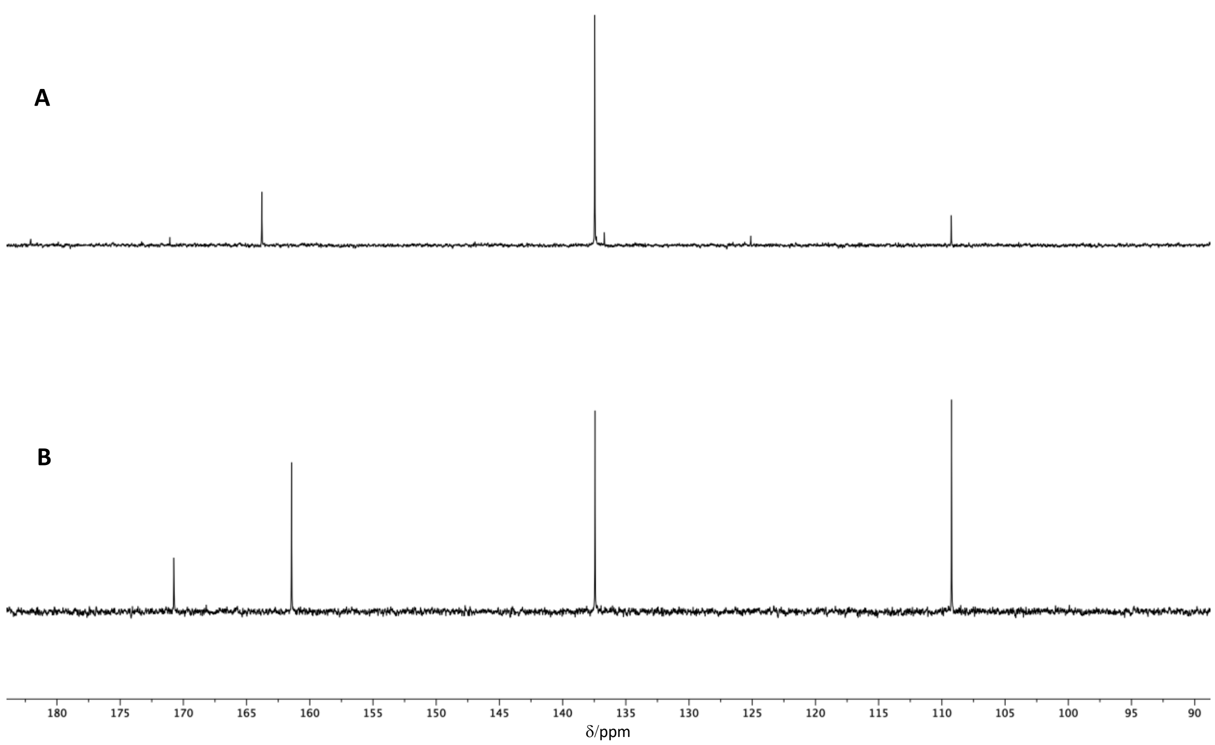

Figure S20 Synthesis of 2-aminothiazole **23** using glycolaldehyde-1-^{13}C **1-**$^{13}C1$. A – ^{13}C NMR spectrum of the crude reaction mixture from Figure S19, spectrum A; B – ^{13}C NMR spectrum of a commercial sample of **23** in bicarbonate buffer at pH 9.2 (δ 170.8, 137.4, 109.2. Peak at 161 ppm is due to bicarbonate buffer). As was observed in the ^{1}H NMR spectrum (Figure S19, spectrum A), the more downfield methine signal (137 ppm, spectrum A) has a far higher degree of ^{13}C incorporation than the upfield methine signal (109 ppm, spectrum A). Given the difference in electronegativity between S and N, it was assumed the downfield methine proton and carbon signals would be attached to C4 in **23**. We then obtained commercial 2-amino-5-methylthiazole and compared its ^{13}C NMR spectrum with that of **23** (Figure S21).

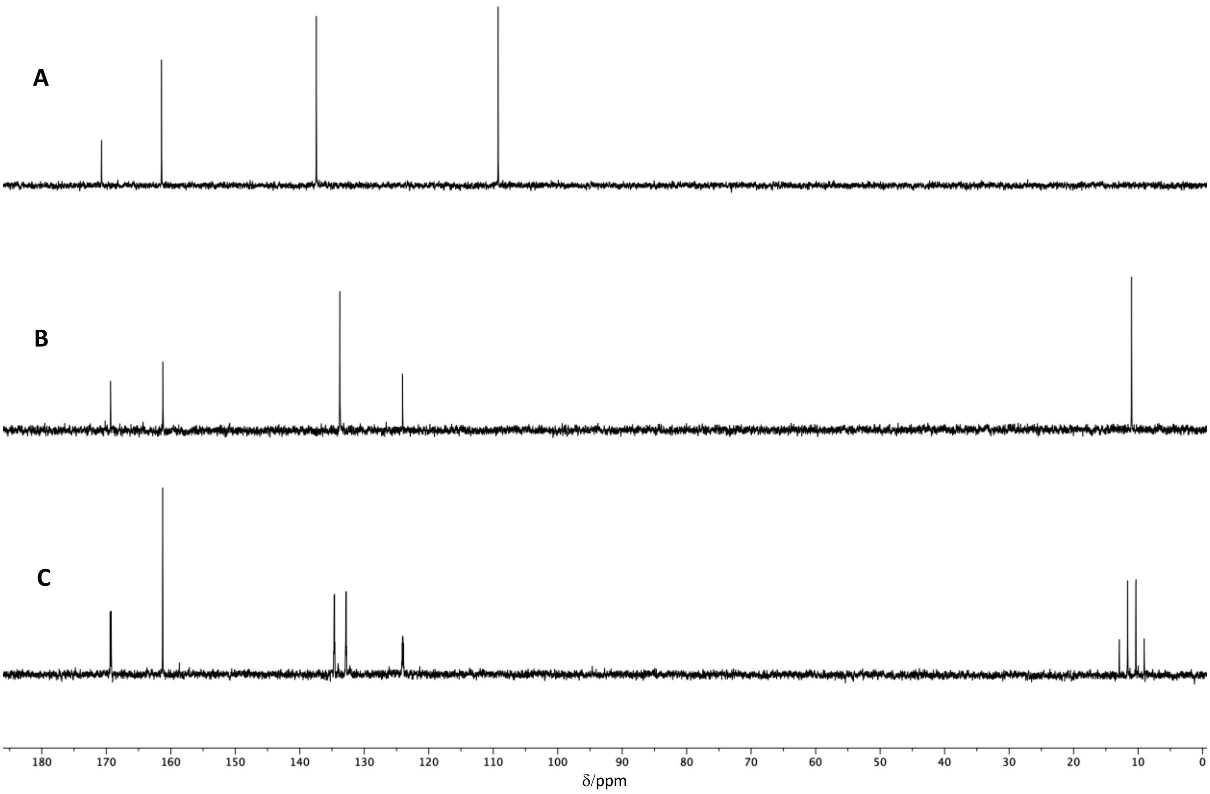

Figure S21 Comparison of 2-aminothiazole **23** and 2-amino-5-methylthiazole ^{13}C NMR spectra. A – ^{13}C NMR Spectrum of a commercial sample of **23** in bicarbonate buffer at pH 9.2 (δ 170.8, 137.4, 109.2. The signal at 161 ppm is due to bicarbonate buffer); B – ^{13}C NMR Spectrum of a commercial sample of 2-amino-5-methylthiazole in bicarbonate buffer at pH 9.2 (δ 169.3, 133.8, 124.1, 11.0. The signal at 161 ppm is due to bicarbonate buffer); C – As spectrum B, but the experiment was run with ^{1}H-^{13}C coupling (δ 169.3 (d, J = 15.6), 133.8 (dq, J = 183, 5.5), 124.1 (dq, J = 13.9, 6.9), 11.0 (qd, J = 129.6, 2.2). The signal at 161 ppm is due to bicarbonate buffer). The aromatic signals of **23** (spectrum A) and 2-amino-5-methylthiazole (spectrum B) correlate well, with the exception of the signal at 109 ppm in spectrum A which shifts 15 ppm downfield in spectrum B and would be consistent with substitution of H for an Me group on C5 in 2-amino-5-methylthiazole. This was confirmed by using a ^{1}H-^{13}C coupling NMR experiment (spectrum C) in which the signal at 124 ppm is shown to be quaternary *i.e.* no $^{1}J_{CH}$ coupling, and, as expected, the more downfield carbon signal at 134 ppm must be that attached to nitrogen. This implies that the signal at 137 ppm in spectrum A correlates to C4 of **23** and the proton signal in the ^{1}H NMR at 6.96 ppm (Figure S19, spectrum A) is that attached to C4.

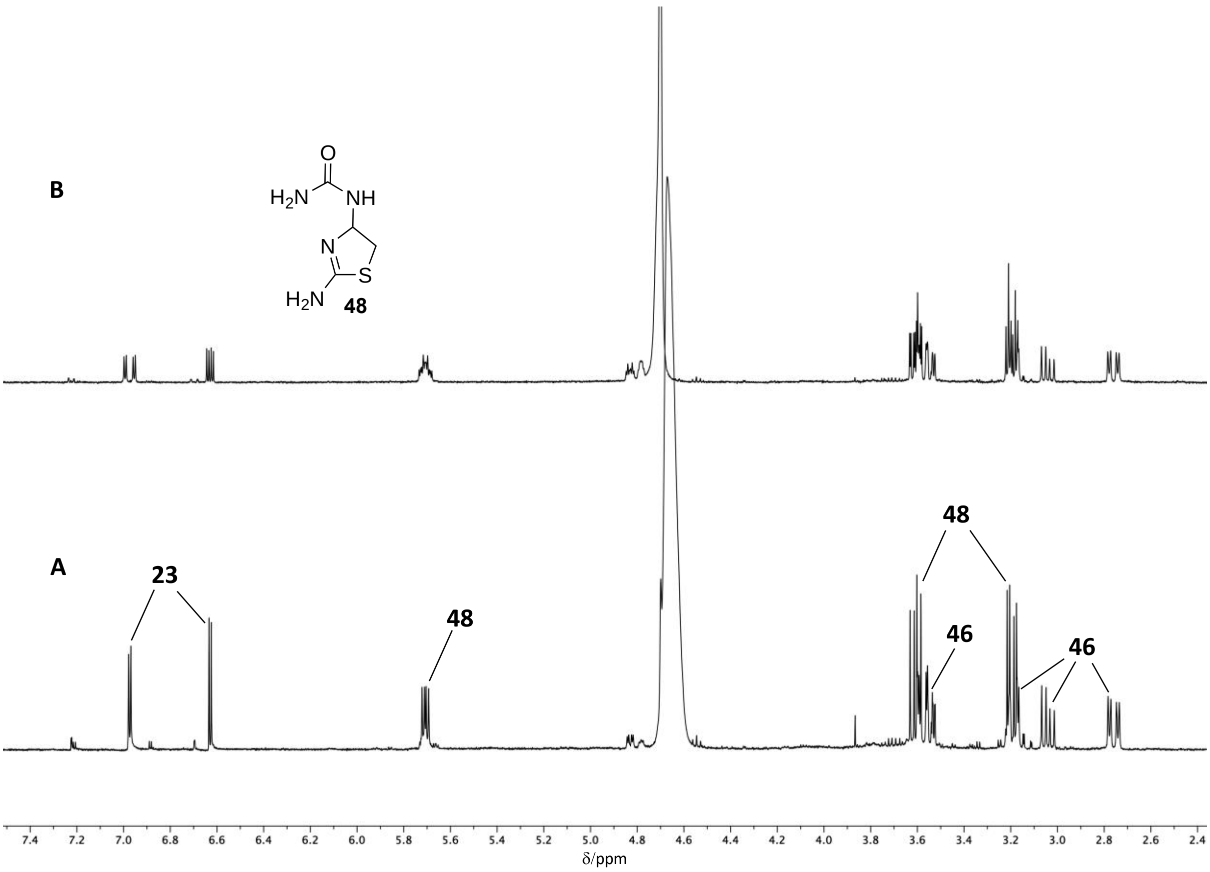

Figure S22 Formation of 2-aminothiazole **23** using NH_2CN or $NH_2{}^{13}CN$. To an Eppendorf tube was charged $NaSH.xH_2O$ (> 60%, 19 mg, 0.200 mmol), degassed 10% D_2O in H_2O (1 mL) and $NaHCO_3$ (34 mg, 0.400 mmol). The pH was adjusted to 9.2 and the volume made up to 2 mL, then **1** (3 mg, 0.050 mmol) and NH_2CN (6 mg, 0.150 mmol) or $NH_2{}^{13}CN$ (7 mg, 0.150 mmol) were added. The vessel was sealed and heated to 60 °C for 3 h, after which a portion was removed and examined by ^1H NMR spectroscopy. A – ^1H NMR Spectrum of the crude reaction mixture using NH_2CN; B – ^1H NMR Spectrum of the crude reaction mixture using $NH_2{}^{13}CN$. The ABX system and chemical shifts seen in spectrum A are consistent with the proposed thiazoline **48** [(D_2O/H_2O) δ 5.71 (dd, J = 6.9, 4.3, 1 H), 3.61 (dd, J = 11.6, 6.9, 1 H), 3.19 (dd, J = 11.6, 4.3, 1 H)]. When ^{13}C-labelled cyanamide was used in the reaction (spectrum B), the pair of double doublets (3.6 and 3.2 ppm) arising from the CH_2 group experienced an additional coupling, giving a doublet of double doublets [δ 3.61 (J = 11.6, 6.9, 2.1)] and a doublet of apparent triplets [δ 3.19 (J = 11.5, 4.3)]. This is also consistent with the proposed structure of **48**, as a ^{13}C-labelled urea group of **48** (4 bond) would be unlikely to couple to the CH_2 group, whereas a ^{13}C-labelled thioisourea group (3 bond) would. The signal at 5.7 ppm in spectrum B has become a multiplet in which there are more than 8 peaks, consistent with the methine proton of **48** coupling to two quaternary ^{13}C-labelled carbons (both 3 bond couplings).

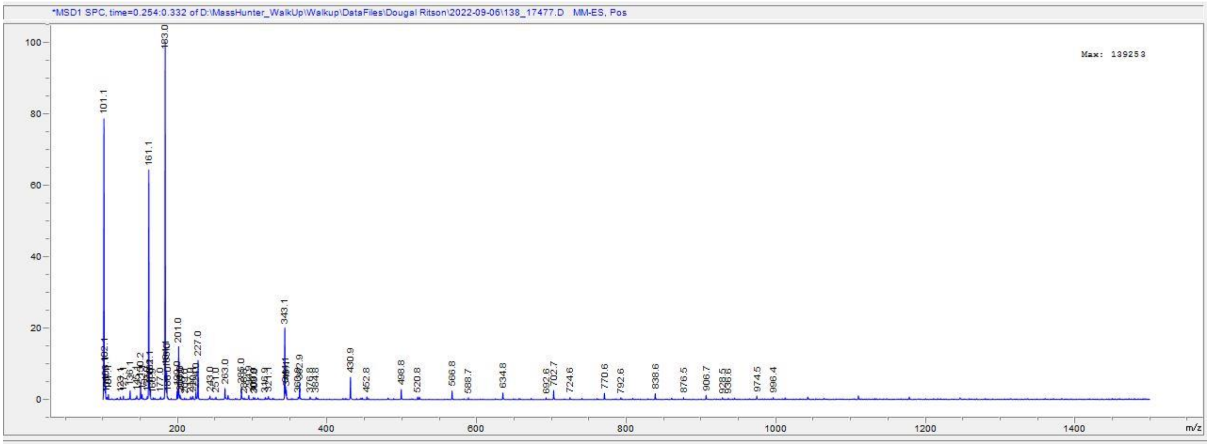

Figure S23 Mass spectrum obtained after LC-MS (ESI(+)) of the crude reaction mixture following the procedure described in Figure S17 after 7 days heating. Thiazoline **48**, M_r = 160.

X-Ray Crystallography

X-ray data were collected for **20** and **46** on a Bruker D8-QUEST diffractometer, equipped with an Incoatec IμS Cu microsource (λ = 1.5418 Å) and a PHOTON-III detector operating in shutterless mode. The crystal temperature was held at 180(2) K using an Oxford Cryosystems open-flow N_2 Cryostream. The control and processing software was Bruker *APEX4* (ver. 2021.4-0). Structures were solved using SHELXT (G. M. Sheldrick, *Acta Cryst. Sect. A*, 2015, **71**, 3–8) and refined using SHELXL (G. M. Sheldrick, *Acta Cryst. Sect. C*, 2015, **71**, 3–8). For **46**, the H atoms of the NH and NH_2 groups were located in the difference Fourier map and refined with isotropic displacement parameters. The N-H distances were restrained to 0.88(1) Å and the H···H distance of the NH_2 group was restrained to 1.51(1) Å.

Summary of crystal and refinement data

	**20**	**46**
CCDC number	2191359	2191360
Cambridge data number	AB_B1_0051	AB_B2_0020
Chemical formula	$C_8H_3N_5$	$C_6H_{12}N_4O_2S_2$
Formula weight	169.15	236.32
Temperature / K	180(2)	180(2)
Crystal system	monoclinic	monoclinic
Space group	C 2/m	P 2_1/c
a / Å	11.1520(5)	6.7518(4)
b / Å	6.1014(2)	9.2109(5)
c / Å	12.1307(5)	8.3592(5)
alpha / °	90	90
beta / °	111.169(2)	97.329(2)
gamma / °	90	90
Unit-cell volume / $Å^3$	769.71(5)	515.61(5)
Z	4	2
Calc. density / g cm^{-3}	1.460	1.522
F(000)	344	248
Radiation type	CuKα	CuKα
Absorption coefficient / mm^{-1}	0.824	4.572
Crystal size / mm^3	0.20 x 0.20 x 0.04	0.20 x 0.15 x 0.06
2-Theta range / degrees	7.82–132.94	13.22–133.16
Completeness to max 2-theta	0.985	0.997
No. of reflections measured	2728	6573
No. of independent reflections	742	901
R(int)	0.0326	0.0302
No. parameters / restraints	79 / 0	76 / 4
Final R1 values ($I > 2\sigma(I)$)	0.0384	0.0574
Final wR(F^2) values (all data)	0.1027	0.1453
Goodness-of-fit on F^2	1.189	1.079
Largest difference peak & hole / e $Å^{-3}$	0.242, –0.319	1.030, –0.590

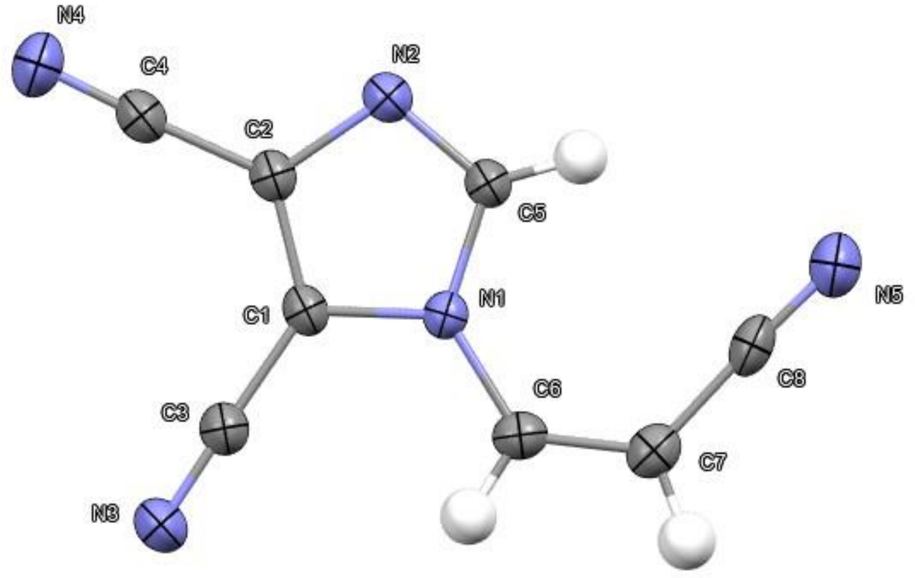

Figure S24. Displacement ellipsoid plot (50% probability) for **20**.

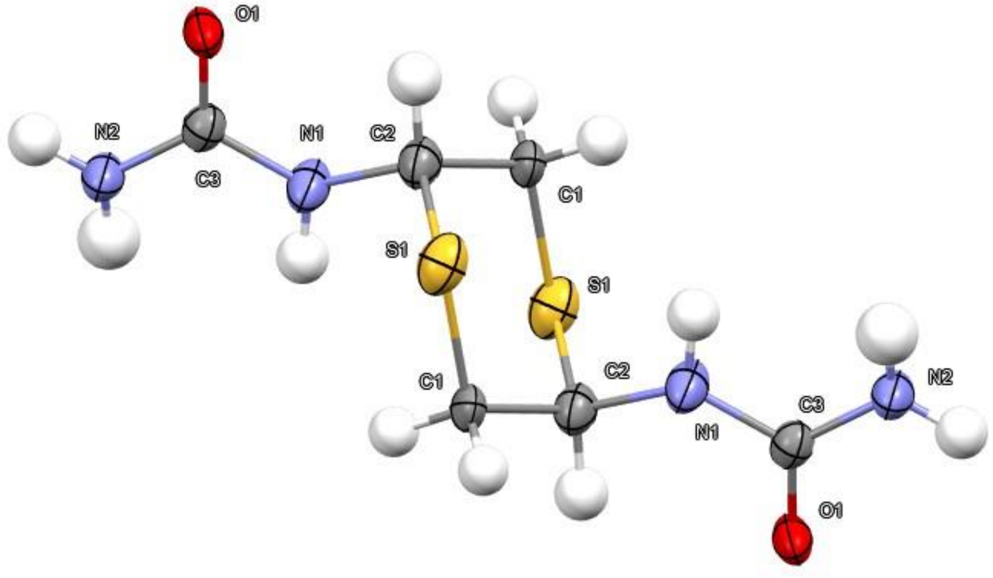

Figure S25. Displacement ellipsoid plot (50% probability) for **46**.

References

1. Xiang, Y.-B.; Drenkard, S.; Baumann, K.; Hickey, D.; Eschenmoser, A. Chemie von α-aminonitrilen. *Helv. Chim. Acta* **1994**, *77*, 2209-2250.
